# *LTK* mutations responsible for resistance to lorlatinib in non-small cell lung cancer harboring *CLIP1-LTK* fusion

**DOI:** 10.1038/s42003-024-06116-6

**Published:** 2024-04-04

**Authors:** Shunta Mori, Hiroki Izumi, Mitsugu Araki, Jie Liu, Yu Tanaka, Yosuke Kagawa, Yukari Sagae, Biao Ma, Yuta Isaka, Yoko Sasakura, Shogo Kumagai, Yuta Sakae, Kosuke Tanaka, Yuji Shibata, Hibiki Udagawa, Shingo Matsumoto, Kiyotaka Yoh, Yasushi Okuno, Koichi Goto, Susumu S. Kobayashi

**Affiliations:** 1 Department of Thoracic Oncology, National Cancer Center Hospital East, Kashiwa, 277-8577 Japan; 2 Graduate School of Medicine, Kyoto University, Shogoin-Kawaharacho, Sakyo-ku, Kyoto 606-8507 Japan; 3 Division of Translational Genomics, Exploratory Oncology Research and Clinical Trial Center, National Cancer Center, Kashiwa, 277-8577 Japan; 4 RIKEN Center for Computational Science, Kobe, Hyogo 650-0047 Japan; 5 Division of Cancer Immunology, Research Institute/Exploratory Oncology Research & Clinical Trial Center, National Cancer Center, Kashiwa, 277-8577 Japan; 6 Department of Integrated Biosciences, Graduate School of Frontier Sciences, The University of Tokyo, Kashiwa, 277-8561 Japan; 7 Department of Medicine, Beth Israel Deaconess Medical Center, Harvard Medical School, Boston, MA 02215 USA

**Keywords:** Oncogenes, Non-small-cell lung cancer

## Abstract

The *CLIP1-LTK* fusion was recently discovered as a novel oncogenic driver in non-small cell lung cancer (NSCLC). Lorlatinib, a third-generation ALK inhibitor, exhibited a dramatic clinical response in a NSCLC patient harboring *CLIP1-LTK* fusion. However, it is expected that acquired resistance will inevitably develop, particularly by *LTK* mutations, as observed in NSCLC induced by oncogenic tyrosine kinases treated with corresponding tyrosine kinase inhibitors (TKIs). In this study, we evaluate eight *LTK* mutations corresponding to *ALK* mutations that lead to on-target resistance to lorlatinib. All *LTK* mutations show resistance to lorlatinib with the L650F mutation being the highest. In vitro and in vivo analyses demonstrate that gilteritinib can overcome the L650F-mediated resistance to lorlatinib. In silico analysis suggests that introduction of the L650F mutation may attenuate lorlatinib-LTK binding. Our study provides preclinical evaluations of potential on-target resistance mutations to lorlatinib, and a novel strategy to overcome the resistance.

## Abstract (web summary)

A novel strategy using gilteritinib can overcome resistance to lorlatinib in non-small cell lung cancer with *CLIP1-LTK*-L650F mutation.

## Introduction

The discovery of several actionable oncogenic drivers in non-small cell lung cancer (NSCLC) and the development of corresponding targeted therapies have changed the treatment strategy, leading to great improvement in patient outcome^1^. The *CLIP1- LTK* fusion is identified as a novel oncogenic driver in NSCLC using a large-scale lung cancer genome screening platform (LC-SCRUM-Asia; UMIN000036871)^2,3^. The CLIP1-LTK fusion protein constitutively activates LTK and its downstream signaling molecules, including AKT and ERK^4^, resulting in cell proliferation and the suppression of apoptosis. This fusion gene is present in 0.4% of NSCLC and is mutually exclusive of other known oncogenic drivers. Interestingly, ALK-tyrosine kinase inhibitors (TKIs), especially lorlatinib, a third-generation ALK-TKI, were effective in cells expressing the *CLIP1-LTK* fusion in vitro and in vivo. The rationale for the use of ALK-TKIs is based on the fact that LTK and ALK share nearly 80% protein sequence identity in their kinase domains, and most ALK-TKIs demonstrate LTK inhibition at ALK-inhibitory concentrations^5,6^. Notably, we also demonstrated that lorlatinib exhibits a dramatic and durable response in a patient with NSCLC harboring *CLIP1-LTK* fusion. However, despite the remarkable efficacy of lorlatinib against NSCLC patients harboring *CLIP1-LTK*, acquired resistance to lorlatinib will inevitably develop, as observed in NSCLC induced by oncogenic tyrosine kinases treated with corresponding TKIs^7–11^. Therefore, it is essential to identify the potential resistance mechanisms of lorlatinib in *LTK* fusion-positive NSCLC and establish effective treatment strategies to overcome this resistance. The mechanism of lorlatinib resistance in *LTK* fusion-positive NSCLC is yet to be elucidated. In general, resistance mechanisms against targeted therapies are divided into three groups: (1) on-target gene alterations^12,13^, (2) off-target mechanisms such as the upregulation of alternative bypass pathways, including *MET* amplifications^14,15^, and (3) histological transformations^16,17^. Among these resistance mechanisms, on-target gene alterations account for 50-70% of patients treated with respective targeted therapies^13,18^.

In this study, we demonstrated that *LTK* mutations, especially the L650F mutation, potentially confer resistance to lorlatinib treatment, and that L650F-mediated resistance to lorlatinib can be overcome by gilteritinib.

## Results

### Lorlatinib is predicted to bind to LTK and ALK

**Fig. 1:**
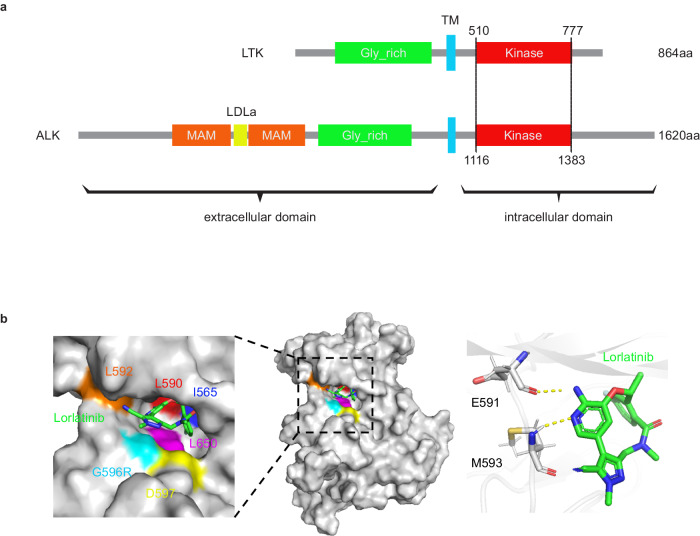
Lorlatinib is predicted to bind to LTK and ALK. **a** Schematic representation of LTK and ALK protein. LTK and ALK are formed with three regions: extracellular, transmembrane, and intracellular region. They consist of 864 amino acids (aa) and 1620 aa respectively. MAM, meprin, A-5 protein, and receptor protein-tyrosine phosphatase μ domain; LDLa, low-density lipoprotein class A motif; Gly_rich, glycine rich region; TM, transmembrane region. **b** The lorlatinib-binding mode for the LTK kinase domain. The protein is depicted by a surface model (I565, blue; L590, red; L592, orange; G596, cyan; D597, yellow; L650, magenta; others, gray) and lorlatinib is depicted by sticks (C, green; N, blue; and O, red). An enlarged view of the ATP-binding pocket is shown in the left panel. In the right panel, hydrogen bonds between LTK residues (E591 and M593) and lorlatinib are shown as dashed yellow lines.

*LTK* and *ALK* belong to the insulin receptor subfamily of receptor tyrosine kinases, which consist of an extracellular region, transmembrane region, and intracellular region. The kinase domain of LTK and ALK contains 268 amino acids (Fig. 1a). Intriguingly, LTK and ALK exhibit 79% amino acid homology in their respective kinase domains^19^, and lorlatinib inhibits LTK at ALK-inhibitory concentrations^5^.

We first evaluated the binding affinity of lorlatinib to LTK proteins using in silico approaches to further support the efficacy of lorlatinib in *CLIP1-LTK* fusion-expressing cells^2^. Molecular dynamics (MD) simulations indicated that lorlatinib fitted into the ATP-binding pocket of LTK and was stabilized by hydrogen bonds with backbone amides of E591 (corresponding to E1197 in ALK) and M593 (corresponding to M1199 in ALK) (Fig. 1b).　In addition, the estimated LTK-lorlatinib binding free energy (ΔG) of −11.6 ± 0.8 kcal/mol is similar to that between ALK and lorlatinib (−14.3 ± 1.3 kcal/mol)^9^. These results suggest that lorlatinib binds to LTK in a manner similar to ALK, further supporting the efficacy of lorlatinib in tumors expressing *CLIP1-LTK* fusion^2^.

### Conserved and homologous sequence of LTK/ALK in the kinase domain responsible for TKI resistance

**Fig. 2:**
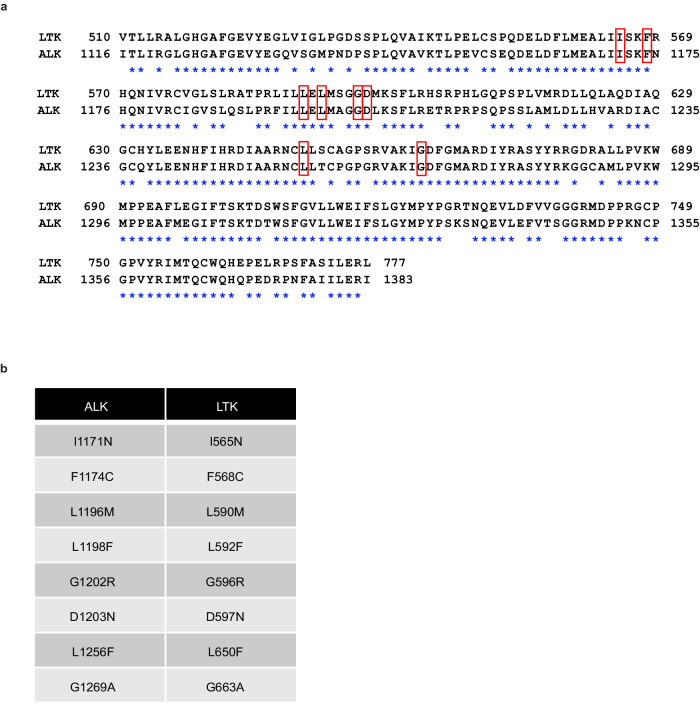
*LTK* mutations are analogous to *ALK* resistant mutations. **a** Amino acid sequences between full LTK and ALK kinase domain. Asterisks represent all the conserved amino acid residues across LTK and ALK. Their homological mutations are surrounded with red boxes. **b** *LTK* and *ALK* corresponding mutations.

The most common resistance mechanism to genotype-matched therapy is caused by acquired genetic alterations in the on-target gene, including gatekeeper or solvent-front mutations^12,13,20,21^. Various ALK-TKI resistance mutations have been identified in *ALK* fusion-positive NSCLC, most of which occur in the ALK kinase domain. Among these kinase domains, eight residues (I1171, F1174, L1196, L1198, G1202, D1203, L1256, G1269) are responsible for lorlatinib resistance in *ALK* fusion-positive NSCLC in clinical setting and/or experimental models^7,9,13,22^. As LTK and ALK exhibit 79% amino acid homology in their respective kinase domains (Fig. 2a), we hypothesized that corresponding mutations to these *ALK*-acquired mutations may emerge in *LTK* fusion-positive cells treated with lorlatinib. Indeed, all these residues are conserved in the LTK protein (Fig. 2a). Thus, we hypothesized that *LTK* mutations analogous to *ALK* mutations could emerge, resulting in lorlatinib resistance (Fig. 2b).

### Analogous *LTK* mutations show lorlatinib resistance

**Fig. 3:**
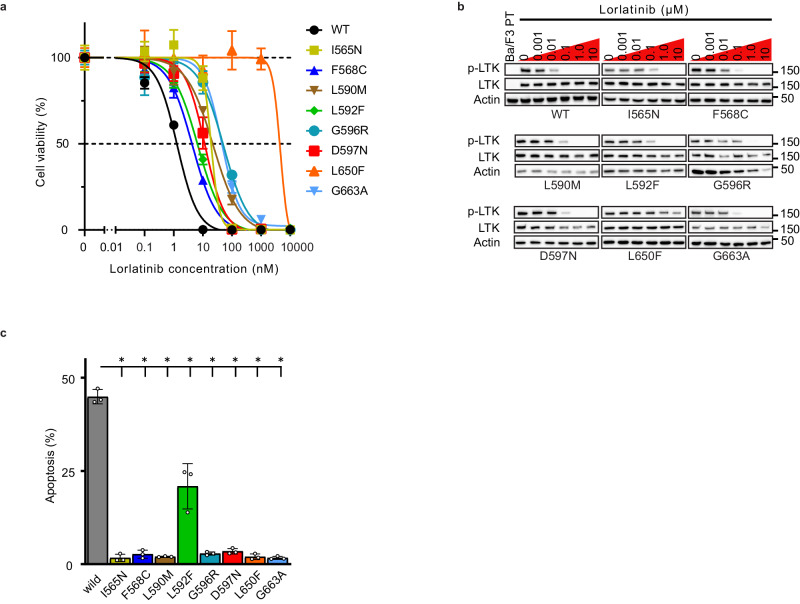
*LTK* mutations are associated with resistance to lorlatinib. **a** Cell viability curves for Ba/F3 cells expressing WT *CLIP1-LTK* and mutant *CLIP1-LTK* treated with lorlatinib at the indicated concentrations for 48 h. Cell viability was evaluated using Cell Counting Kit-8. Error bars are indicated as mean ± SD from three independent experiments. **b** The attenuation of LTK phosphorylation in Ba/F3 cells expressing *CLIP1-LTK* with eight mutations treated with lorlatinib at the increasing concentrations for 24 h. Cell extracts were analyzed by western blotting assay using the indicated antibodies. p-LTK, phospho-LTK. **c** The percentage of apoptosis in Ba/F3 cells expressing *CLIP1-LTK* with eight mutations treated with 10 nM lorlatinib for 24 h. The cells were stained with AnnexinV and propidium iodide. Apoptotic cells were then measured by flow cytometry. Error bars are indicated as mean ± SD from three independent experiments. ^*^p < 0.001 (Dunnett’s test).

We then established Ba/F3 and NIH3T3 cells expressing CLIP1-LTK fusion proteins with the aforementioned *LTK* mutations to clarify the impact of these mutations on sensitivity to lorlatinib, as well as other targeted agents. Cell viability assays using Ba/F3 cells expressing WT *CLIP1-LTK* or each *CLIP1-LTK* mutation revealed that Ba/F3 cells expressing mutant *CLIP1-LTK* were less sensitive to lorlatinib compared with those expressing WT *CLIP1-LTK* (Fig. 3a). The western blotting assay also showed that the effect of lorlatinib on LTK tyrosine phosphorylation was attenuated in Ba/F3 cells expressing mutant *CLIP1-LTK* compared with Ba/F3 cells expressing WT *CLIP1-LTK*. Ten nM of lorlatinib or higher inhibited the LTK tyrosine phosphorylation of Ba/F3 cells expressing WT *CLIP1-LTK*, whereas that of *CLIP1-LTK* with kinase mutations was not inhibited by 10 nM of lorlatinib (Fig. 3b). In particular, the L650F mutation was the most resistant to lorlatinib in terms of inhibition of cell proliferation with IC_50_ value as well as LTK phosphorylation (Fig. 3a, b).

Furthermore, cell apoptosis was evaluated in Ba/F3 cells expressing WT or mutant *CLIP1-LTK* treated with lorlatinib. Ba/F3 cells expressing *CLIP1-LTK*-L592F were more susceptible to lorlatinib than those expressing other *CLIP1-LTK* mutations possibly due to differences in cell growth rates. However, apoptosis was significantly suppressed in all *CLIP1-LTK* mutant Ba/F3 cells compared to those expressing WT *CLIP1-LTK* (Fig. 3c). These results suggested that these *LTK* mutations are resistant to lorlatinib-induced LTK kinase inhibition and cell apoptosis.

### Cell viability profiles of ALK inhibitors

**Fig. 4:**
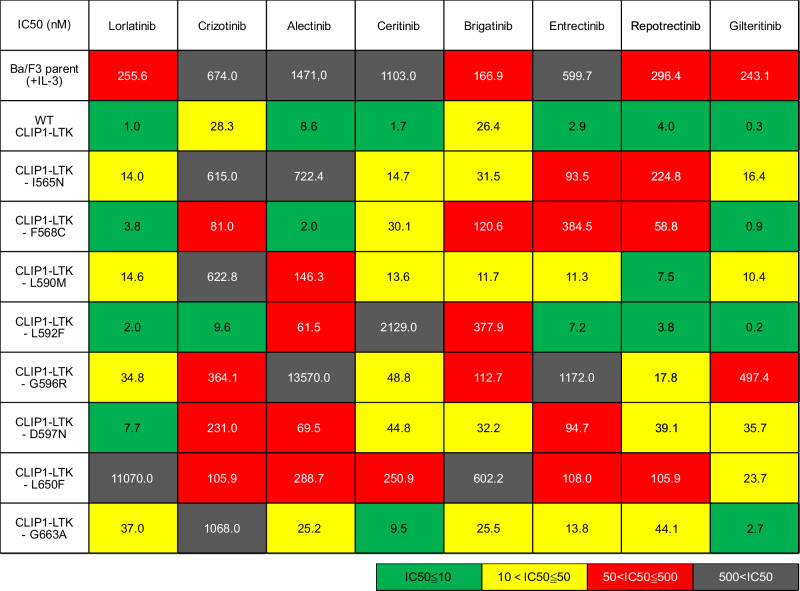
IC_50_ values of eight compounds in Ba/F3 cells expressing indicated *LTK* mutations. Parental Ba/F3 cells and Ba/F3 cells expressing WT *CLIP1-LTK* and mutant *CLIP1-LTK* were treated with the eight indicated inhibitors at several concentrations for 48 h. Cell viability was evaluated using the Cell Counting Kit-8. The mean IC_50_ values are shown.

Next, we explored potential compounds that could overcome lorlatinib resistance mediated by these *LTK* mutations and evaluated the sensitivity of the following compounds: lorlatinib, crizotinib, alectinib, ceritinib, brigatinib, entrectinib, repotrectinib, and gilteritinib. To compare the sensitivity to each compound in Ba/F3 cells expressing WT or mutant *CLIP1-LTK*, the IC_50_ values of the compounds were determined in a cell viability assay. The IC_50_ values of these eight compounds to Ba/F3 cells expressing WT *CLIP1-LTK* or other mutations are shown in Fig. 4. All *LTK* mutations showed resistance to lorlatinib, with IC_50_ values of lorlatinib ranging 2.0 to 11,070 nM, which were higher than that of WT *CLIP-LTK* (1.0 nM). Notably, the IC_50_ of lorlatinib against Ba/F3 cells expressing *CLIP1-LTK*-L650F was 11,070 nM, which was the highest among the *LTK* mutations tested in this study, while the IC_50_ of gilteritinib against Ba/F3 cells expressing *CLIP1-LTK*-L650F was 23.7 nM, which was the lowest among the tested compounds.

Moreover, gilteritinib can also inhibit Ba/F3 expressing WT *CLIP1-LTK*, with IC_50_ value of 0.3 nM, which is consistent with a previous report demonstrating gilteritinib shows LTK inhibition at a similar concentration^23^. We also explored potential compounds that could overcome resistance by G596R, L650F, and G663A, which may induce high-level resistance to lorlatinib. The western blotting assay showed that repotrectinib inhibited LTK phosphorylation of CLIP1-LTK-G596R at a concentration of 100 nM or higher, whereas lorlatinib at a concentration of 1 µM or higher was required to achieve this (Supplementary Fig. 1). Similarly, gilteritinib successfully inhibited LTK phosphorylation of CLIP1-LTK-L650F or G663A at 100 nM, whereas lorlatinib did so at concentrations of 1 µM or higher (Supplementary Fig. 1). In addition, we evaluated a combination of repotrectinib and gilteritinib using Ba/F3 cells expressing WT or mutant *CLIP1-LTK*. The combination significantly enhanced suppression of cell viability compared to repotrectinib or gilteritinib alone in most cells. However, such effect was not observed in Ba/F3 cells expressing WT *CLIP1-LTK* or L650F (Supplementary Fig. 2).

### Gilteritinib overcomes lorlatinib resistance by *LTK* L650F mutation in vitro and in vivo

**Fig. 5:**
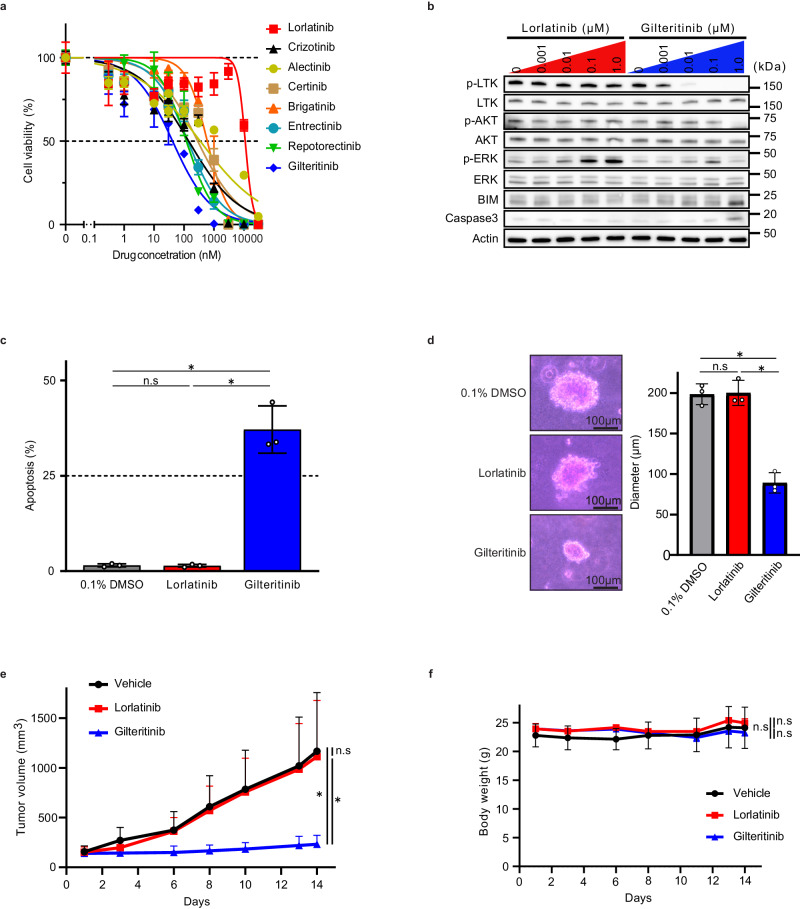
Gilteritinib is potent in overcoming lorlatinib resistance by *CLIP1-LTK*-L650F. **a** Cell viability curves for Ba/F3 cells expressing *CLIP1-LTK*-L650F treated with the indicated compounds at the increasing concentrations for 48 h. Cell viability was evaluated using Cell Counting Kit-8. Error bars are indicated as mean ± SD from three independent experiments. **b** Western blotting showing LTK and its downstream signaling molecules in Ba/F3 cells expressing *CLIP1-LTK*-L650F. The cells were treated with lorlatinib and gilteritinib at the indicated concentrations for 16 h. Cell extracts were analyzed by western blotting assay using the indicated antibodies. p-LTK, phospho-LTK; p-AKT, phospho-AKT; p-ERK, phospho-ERK. **c** The percentage of apoptosis in Ba/F3 cells expressing *CLIP1-LTK*-L650F treated with 0.1% DMSO, lorlatinib (1 μM) and gilteritinib (1 μM) for 24 h. The cells were stained with AnnexinV and propidium iodide. Apoptotic cells were then measured by flow cytometry. Error bars are indicated as mean ± SD from three independent experiments. *p < 0.001; n.s, not significant (Turkey’s test). **d** The diameters of NIH3T3 cells carrying *CLIP1-LTK*-L650F treated with 0.1% DMSO, lorlatinib (1 μM) and gilteritinib (1 μM) for 14 days. Error bars are indicated as mean ± SD from three independent experiments. Scalebars, 100 μm. *p < 0.001; n.s, not significant (Turkey’s test). **e** Inhibition of lorlatinib and gilteritinib against mouse tumors bearing NIH3T3 cells expressing *CLIP1-LTK*-L650F. Mice were treated with either lorlatinib (10 mg/kg once daily), gilteritinib (30 mg/kg once daily) or vehicle control. Error bars are indicated mean ± SD (n = 6 for each group) *p < 0.05; **p < 0.01; n.s, not significant (Turkey’s test) **f** Body weight changes in mice indicated in **e**. Error bars are indicated as mean body weight± SD (*n* = 3 for each group) and statistically analyzed by Turkey’s test. n.s, not significant.

We further focused on *CLIP1-LTK*-L650F, which was the most resistant strain to lorlatinib in this study. Among the tested compounds, gilteritinib was the most potent in Ba/F3 cells expressing *CLIP1-LTK*-L650F (Fig. 5a). Therefore, we investigated whether gilteritinib could overcome resistance to lorlatinib induced by *CLIP1-LTK*-L650F. The western blotting assay showed that gilteritinib successfully inhibited LTK phosphorylation in Ba/F3 cells expressing *CLIP1-LTK*-L650F. Indeed, at 100 nM, gilteritinib strongly attenuated AKT and ERK phosphorylation, whereas lorlatinib did not. In addition, gilteritinib increased the levels of the stabilized form of BIM and cleaved caspase-3, the hallmark of apoptosis (Fig. 5b). Fluorescence-activated cell sorting (FACS) analysis using annexin V/propidium iodide (PI) staining also confirmed that gilteritinib induced apoptosis in Ba/F3 cells carrying *CLIP1-LTK*-L650F (Fig. 5c). An increase in caspase activity by gilteritinib, but not lorlatinib, also supported the successful induction of apoptosis by gilteritinib (Supplementary Fig. 3).

We subsequently investigated the inhibitory effect of gilteritinib in another cell model, NIH3T3 cells carrying *CLIP1-LTK*-L650F, using a soft agar colony formation assay. The diameter of colonies treated with gilteritinib was significantly smaller than that treated with lorlatinib or dimethylsulfoxide (DMSO), whereas lorlatinib did not inhibit colony formation compared with DMSO (Fig. 5d).

Finally, we tested the activity of gilteritinib against *CLIP1-LTK*-L650F cells using a xenograft model. Consistent with the results of the in vitro experiments, there was no significant difference in tumor size between the lorlatinib and vehicle control groups, suggesting the robust resistance of *CLIP1-LTK*-L650F to lorlatinib. In contrast, gilteritinib significantly inhibited tumor growth compared with the vehicle control or lorlatinib (Fig. 5e). Notably, no significant difference in body weight was detected among these three groups, suggesting that gilteritinib showed less toxicity (Fig. 5f). Collectively, gilteritinib potentially overcame the L650F-mediated resistance to lorlatinib in tumors expressing *CLIP1-LTK*-L650F.

### L650F mutation disturb lorlatinib binding to LTK

**Fig. 6:**
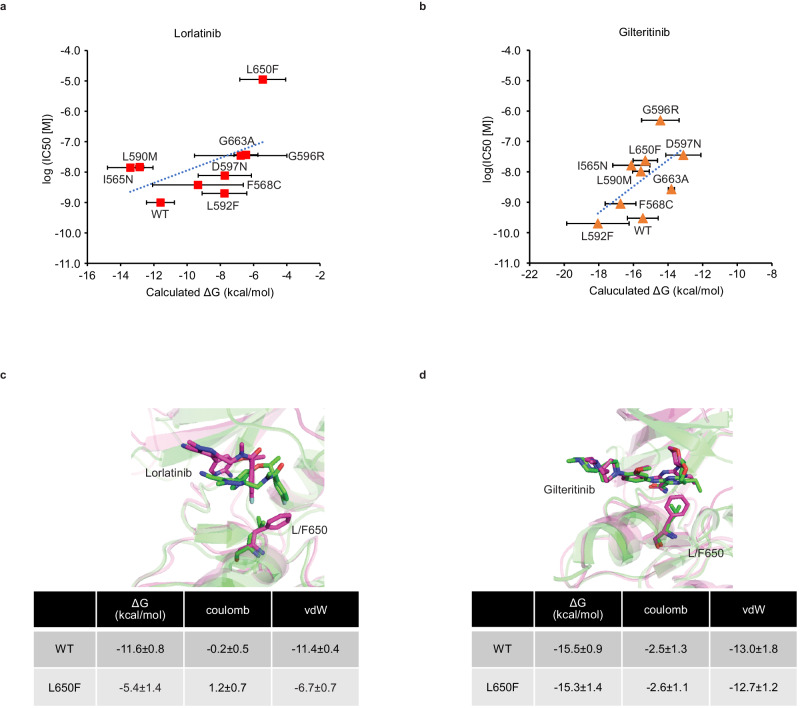
Gilteritinib has potent to overcome lorlatinib resistance by CLIP1-LTK-L650F. The plots of the binding free energy (ΔG) of lorlatinib (**a**) or gilteritinib (**b**) to WT CLIP1-LTK or each mutant CLIP1-LTK against experimental IC_50_ values of the lorlatinib or gilteritinib in Ba/F3 cells expressing the corresponding *CLIP1-LTK* as shown in Fig. 4. These ΔG values were calculated by MP-CAFEE. MD-relaxed structures of **c** lorlatinib or **d** gilteritinib-bound WT CLIP1-LTK (green) and the L650F mutant (magenta). The energetically-stable structure for each LTK–drug complex was extracted from five independent 50 ns MD simulations. The protein backbone is represented by a ribbon diagram, and L/F650 and lorlatinib/gilteritinib are depicted by sticks (C, green/magenta; N, blue; O, red). ΔG values and electrostatic (Coulomb) and van der Waals (vdW) contributions to them are also indicated.

We further explored how these *LTK* mutations affect sensitivity to lorlatinib and gilteritinib. As lorlatinib failed to inhibit LTK phosphorylation in cells with *LTK* mutations, we speculated that these mutations affected LTK-lorlatinib binding. We estimated the binding affinity of lorlatinib against WT CLIP1-LTK and its mutants using the Massively Parallel Computation of Absolute binding Free Energy with well-equilibrated states (MP-CAFEE) method^24^ and found that the IC_50_ of lorlatinib showed in Fig. 4 was well correlated with the LTK-lorlatinib ΔG, with a correlation coefficient (R) of 0.509 (Fig. 6a). We also observed a moderate correlation between the IC_50_ and ΔG values of gilteritinib, with an R of 0.597 (Fig. 6b). These results suggested that decreased LTK-drug binding affinity is a major contributor to mutation-induced drug resistance. For example, the binding affinity of lorlatinib to CLIP1-LTK-L650F (ΔG, −5.4 ± 1.4)　was significantly lower than that of WT CLIP1-LTK (ΔG, −11.6 ± 0.8) due to the loss of intermolecular van der Waals interactions, leading to a large displacement of the drug in the pocket (Fig. 6c). In contrast, no significant difference in the binding affinity of gilteritinib was observed between WT CLIP1-LTK and CLIP1-LTK-L650F, suggesting that this mutation has little effect on gilteritinib binding (Fig. 6d).

## Discussion

This study was the first to explore potential *LTK* resistance alternations against lorlatinib in tumors expressing the CLIP1-LTK fusion protein. We found that all eight *LTK* tested mutations were responsible for lorlatinib resistance, among which the L650F mutation showed the most robust resistance to lorlatinib. We also demonstrated that gilteritinib was an exquisite and potent inhibitor of *CLIP1-LTK*-L650F in in vivo and in vitro experiments.

*LTK* fusion is a rare but actionable oncogenic driver in NSCLC^2^. To clinically develop an effective targeted therapy, an investigator-initiated clinical trial (IIT) of lorlatinib for advanced NSCLC with *LTK* fusion is ongoing using the LC-SCRUM-Asia (jRCT2031220600). However, acquired resistance to lorlatinib inevitably develops despite the expected initial favorable efficacy. To develop more efficient resistance mechanism-matched therapies, we explored the potential mechanism of resistance to lorlatinib in preclinical models. In general, resistance mechanisms against targeted therapies are divided into three groups: 1) on-target gene alterations^7,13^, 2) off-target mechanisms, such as the upregulation of alternative bypass pathways, including *MET* amplifications^14,15^, and 3) histological transformations^16,17^ Among these resistance mechanisms, on-target gene alterations account for 50-70% of patients treated with respective targeted therapies^13^. In this study, we focused on *LTK* mutations among the various resistance mechanisms against ALK-TKIs. However, it is certainly possible that other resistance mechanisms, including off-target mechanisms, such as the upregulation of alternative bypass pathways, can emerge. Along with LC-SCRUM-Asia, we conduct genomic screening for treatment-resistant patients with advanced NSCLC (LC-SCRUM-TRY; UMIN000041957) to identify the resistance mechanisms and support the clinical development of resistance mechanism-matched therapies. We will explore the mechanism of lorlatinib resistance using clinical samples obtained from patients enrolled in the IIT.

In this study, we showed that gilteritinib can inhibit kinase activity of CLIP1-LTK with *LTK* mutations including L650F, as well as WT *CLIP1-LTK*. Gilteritinib may be an alternative option for *LTK* fusion-positive NSCLC as either a first TKI treatment or second TKI after lorlatinib treatment.

Considering similar pattern of drug sensitivity between *ALK* and *LTK*, and the rarity of patients harboring specific resistance mechanisms, basket-type trials of targeted therapy for patients with specific resistance mutations might be useful, for example for L1256F/L650F mutated *ALK/LTK* fusion-positive NSCLC to efficiently develop targeted therapy for rare fusion-positive NSCLC resistant to prior targeted therapies.

In summary, *LTK* mutations analogous to *ALK* mutations were resistant to lorlatinib, with the L650F mutation being the most potent. Our preclinical models demonstrate that gilteritinib may be a promising strategy to overcome L650F-mediated resistance.

## Materials and methods

### Cell lines and reagents

NIH3T3 cells were purchased from American Type Culture Collection (ATCC). Ba/F3, WEHI, and BOSC23 cells were kindly provided by Dr. Daniel G. Tenen (Harvard Medical School). Crizotinib, ceritinib, alectinib, brigatinib, lorlatinib, entrectinib, gilteritinib, and repotrectinib were purchased from Selleck. NIH3T3 cells were maintained in Dulbecco’s Modified Eagle Medium (DMEM) supplemented with 10% fetal bovine serum (FBS), 100 units/ml penicillin, and 100 mg/ml streptomycin (P/S). Parental Ba/F3 cells were maintained in RPMI1640 supplemented with 5% WEHI (as a source of IL-3), 10% FBS, and P/S. Ba/F3 cells expressing *CLIP1-LTK* mutants were maintained in RPMI1640 supplemented with 10% FBS and P/S. All cell lines were routinely tested for mycoplasma infection and negative for mycoplasma infection.

### Construction of plasmid

The MIGR1 retroviral vector harboring CLIP1-LTK fusion protein was constructed as previously described^2^. Plasmids expressing each mutant *CLIP1-LTK* (I565N, F568C, L590M, L592F, G596R, D597N, L650F, and G663A) were generated using the Quick Change Lightning Site-Directed Mutagenesis Kit (Agilent). All the primers used are listed in Supplementary Table 1. The integrity of all constructs was confirmed by Sanger sequencing.

### Viral transduction

Ba/F3 and NIH3T3 cells expressing WT *CLIP1-LTK* fusion or various mutant *CLIP1-LTK* fusions were generated by retroviral transduction as previously described^2^.

### Western blotting

Cells were lysed in sodium dodecyl sulfate (SDS) sample buffer and boiled for 5 min. Lysates were subjected to SDS polyacrylamide gel electrophoresis and blotted onto poly (vinylidene fluoride) (PVDF) membranes (Millipore). The antibodies and dilutions used are listed in Supplementary Table 2. Images were captured using ImageQuant LAS 4000 (GE Healthcare) and analyzed using the ImageJ software (ver. 1.53). All images were assembled, and figures were generated using the Affinity Designer (ver. 1.10.5), and Microsoft PowerPoint 2016 (ver. 2108).

### Cell viability assay

Ba/F3 cells (10,000 cells per well) were seeded in 96-well plates and treated with inhibitors of interest for 48 h, and viability was evaluated using the Cell Counting Kit-8 (Fujifilm). Data were captured using the Spectra Max Paradigm (Molecular Devices) with SoftMax Pro software (ver.7.10). Absorbance was measured at a wavelength of 450 nm. The IC_50_ value was determined using a nonlinear regression model (four parameters) using the GraphPad Prism software (ver. 9.3.1).

### Soft ager formation assay

NIH3T3 cells expressing *CLIP1-LTK*-L650F (30,000 cells per well) were seeded in 6-well plates and treated with the inhibitors of interest for 14 days. Cell images were captured using the BZ-II Viewer software (v. 2.10), and the diameter of the colonies was measured using ImageJ software (ver. 1.53).

### Apoptosis assay

Ba/F3 cells (100,000 cells/well) were seeded in 6-well plates. After they were treated with the inhibitors of interest for 24 h, they were stained with annexin-V and PI using the Annexin V-FITC Apoptosis Detection Kit (Nacalai Tesque). A total of 10,000 cells were captured using FACSDiva software (v. 9.0). FACS data were analyzed using FlowJo software (v. 10.7.1). Gating was conducted to detect single cells and then determined so that there were no annexin V-positive cells in untreated Ba/F3 cells. Cells undergoing apoptosis were defined as annexin V-positive cells.

The Caspase-Glo3/7 Assay System (Promega) was used to evaluate cell apoptosis. Ba/F3 cells (5000 cells/well) were seeded in 96-well plates and treated with the indicated drugs for 12 h. Data were captured using the Spectra Max Paradigm (Molecular Devices) with SoftMax Pro software (ver.7.10). Absorbance was measured at 490 nm.

### Molecular docking

Molecular docking of alectinib, gilteritinib, and lorlatinib with the LTK-tyrosine kinase domain was performed using GOLD 5.5. Standard default settings for the genetic algorithm were used. The structure of the LTK kinase domain was predicted using AlphaFold2^25^. The dominant protonation state at pH 7.0 was assigned to titratable residues. The ATP-binding site was defined to include all atoms within 10 Å of the midpoint of the Leu516 Cα and Gly596 Cα atoms. Alectinib, gilteritinib, and lorlatinib, whose 3D structures were obtained from the crystal structures of ALK-alectinib (PDBID:3AOX), FLT3-gilteritinib (PDBID:6JQR), and ALK-lorlatinib complexes (PDBID:4CLI), respectively, were protonated to form ionization states in solution (net charges of +1, +2, and 0, respectively). After the backbone Cα atoms in LTK were structurally aligned with those in each crystal structure, alectinib, gilteritinib, and lorlatinib were docked into the ATP-binding site in LTK with positional restraints on the benzocarbazole, pyrazinamide, and cyclotetradecine moieties, respectively, assuming that these drugs had a similar binding geometry between LTK and ALK/FLT3. The top-ranked docking pose was extracted and used as the initial structure for MD simulations of the LTK drug complexes.

### MD simulation of wild-type LTK or its mutants in complex with drugs

Each of I565N, F568C, L590M, L592F, G596R, D597N, L650F, and G663A mutations were introduced into the structural model of WT LTK using the MODELER program^26^. According to a previously described procedure^27^, computational systems of LTK-drug complexes were prepared, and MD simulations were carried out. For each LTK mutant, five independent production runs of 50 ns (with different atomic velocities) were performed in a constant number of molecules, pressure, and temperature (NPT) ensemble, where the temperature was maintained at 298 K by stochastic velocity rescaling^28^. A Parrinello-Rahman barostat was used to maintain the pressure at 1 bar^29^, with the temperature and pressure time constants set to 0.1 and 2 ps, respectively. Three sets of 20 ns production runs were performed for the solvated drug system.

The LTK-drug ΔG was calculated using MP-CAFEE, which is one of the chemical free energy perturbation methods^24^. The ΔG for each LTK mutant was computed according to a protocol described in a previous study^30^. The GROMACS 2019 and 2021 programs^31^ were used for the free energy simulations and preceding production runs, respectively.

### Xenograft experiments

The Institutional Animal Care and Use Committee of the National Cancer Center (K20-009) approved all the animal experiments. We have complied with all relevant ethical regulations for animal use. To establish tumor xenografts, NIH3T3 cells transduced with *CLIP1-LTK-*L650F were transplanted into the flanks of athymic nude mice (female, 8-weeks old BALB/cAJcl-*Foxn1*^*nu*^, CLEA Japan). The mice were housed on a 12:12 light/dark cycle, and the temperature was maintained at 24 °C (23–25 °C) and humidity at 49% (40–60%). When the mean tumor volume reached 100-200 mm^3^, mice were randomized into three groups and treated with lorlatinib (10 mg/kg once daily), gilteritinib (30 mg/kg once daily), or vehicle control by oral gavage. Lorlatinib was formulated in 2% DMSO and 30% polyethylene glycol 300 in H_2_O. Gilteritinib was formulated using 0.5% methylcellulose in H_2_O. Tumor volumes (six tumors per group) were calculated using the following formula^32^: 1/2(length × width^2^).

### Statistics and Reproducibility

The group size was based on previous experience. Unless otherwise noted, each experiment was repeated three or more times with similar results. One-way ANOVA and post-hoc analysis, including Dunnett’s test and Tukey’s test, were used to determine statistical significance among more than three groups. All statistical analyses were conducted on data from three or more biologically independent experimental replicates using the GraphPad Prism software (ver. 9.3.1). Statistical significance was set at *p* < 0.05.

### Reporting summary

Further information on research design is available in the Nature Portfolio Reporting Summary linked to this article.

### Supplementary information

Supplementary Information

Description of Additional Supplementary Files

Supplementary Data 1

Reporting Summary

## Data Availability

The sequence data used in this study are publicly available in the National Center for Biotechnology Information (https://www.ncbi.nlm.nih.gov/). The protein structure data are publicly available in RCSB Protein Data Bank (https://www.rcsb.org/). The uncropped western blotting images were exhibited in Supplementary Fig. 4. The gating strategy was exhibited in Supplementary Fig. 5. Source data behind the graphs can be found in the Supplementary Data file. All other data are available through the corresponding author (Susumu S. Kobayashi: skobayas@bidmc.harvard.edu).
