# Supplementary material for: LTK mutations responsible for resistance to lorlatinib in non-small cell lung cancer harboring CLIP1-LTK fusion
Source: Commun Biol. 2024 Apr 4;7:412. doi: 10.1038/s42003-024-06116-6 (PMC10995188; doi:10.1038/s42003-024-06116-6)

Supplementary Table.1 Oligonucleotides used in this study

Name	Direction	Sequence
CLIP1-LTK-I565N (t1694a)	F	GGCGAAACTTGCTGTTGATGAGGGCCTCC
	R	GGAGGCCCTCATCAACAGCAAGTTTCGCC
CLIP1-LTK-F568C (g1703t)	F	TCTGATGGCGACACTTGCTGATGATGAGGGCCT
	R	AGGCCCTCATCATCAGCAAGTGTCGCCATCAGA
CLIP1-LTK-L590M (c1768a)	F	CCAGACATCAGTTCCATCAGAATGAGGCGAGGG
	R	CCCTCGCCTCATTCTGATGGAACTGATGTCTGG
CLIP1-LTK-L592F (c1774t_g1776c)	F	TGTCCCCTCCAGACATGAATTCCAGCAGAATGAGGC
	R	GCCTCATTCTGCTGGAATTCATGTCTGGAGGGGACA
CLIP1-LTK-G596R (g1786a)	F	AACTCTTCATGTCCCTTCCAGACATCAGTTCCAG
	R	CTGGAACTGATGTCTGGAAGGGACATGAAGAGTT
CLIP1-LTK-D597N (g1789a)	F	CAGGAAACTCTTCATGTTCCCTCCAGACATCAGTT
	R	AACTGATGTCTGGAGGGAACATGAAGAGTTTCCTG
CLIP1-LTK-L650F (c1948t_g1950c)	F	CAGCGCAGCTCAGGAAGCAGTTCCGGGCG
	R	CGCCCGGAACTGCTTCCTGAGCTGCGCTG
CLIP1-LTK-G663A (g1988c)	F	GCCATCCCAAAGTCCGCAATCTTGGCCACTC
	R	GAGTGGCCAAGATTGCGGACTTTGGGATGGC

Supplementary Table.2 Antibodies used in this study

Name	Company	Catalog number	Dilutiuon
phospho ALK/LTK (pTyr1278/672)	Cell Signaling Technology	6941	1:1000
phospho Akt (pS473)	Cell Signaling Technology	4058	1:1000
phospho Erk1/2 (pT202/pY204)	Cell Signaling Technology	9106	1:1000
total LTK	Abcam	ab129155	1:1000
total Akt	Cell Signaling Technology	4685	1:1000
total Erk1/2	Cell Signaling Technology	9102	1:1000
BIM	Cell Signaling Technology	2819	1:1000
Cleaved caspase-3 (Asp175)	Cell Signaling Technology	3075	1:1000
β-actin	Cell Signaling Technology	4970	1:5000
Anti-rabbit IgG HRP-linked Secondary Antibody	Cell Signaling Technology	7074	1:5000
Anti-mouse IgG, HRP-linked Antibody	Cell Signaling Technology	7076	1:5000

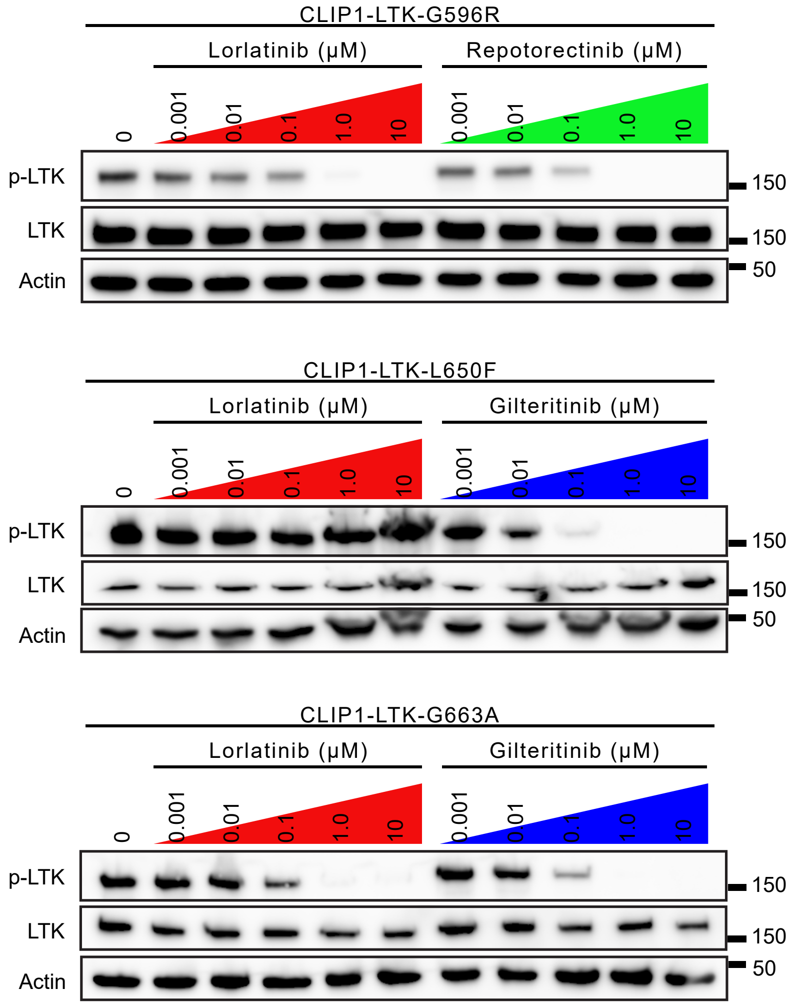

Supplementary Figure.1 Attenuation of LTK phosphorylation in Ba/F3 cells expressing mutant *CLIP1-LTK* (G596R, L650F, and G663A) treated with the indicated compounds at increasing concentrations for 3h. The cell extracts were analyzed by western blotting using the indicated ntibodies.

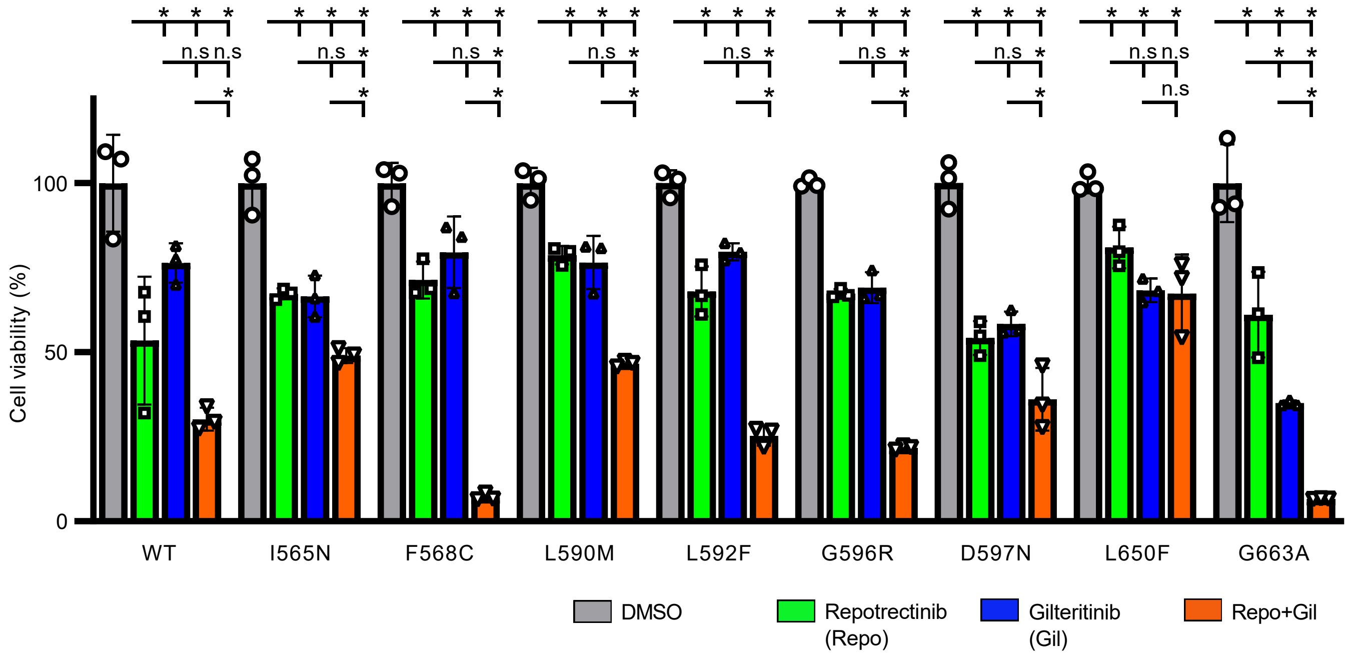

Supplementary Figure.2 Cell Viability for Ba/F3 cells expressing *CLIP1-LTK* with eight mutations treated with 10nM repotrectinib, 10nM gilteritinib and the combination of 10nM repotrectinib plus 10nM gilteritinib for 48 h. Cell viability was evaluated using Cell Counting Kit-8. Error bars are presented as mean± SD. * p<0.05 (Dunnett's test); n.s, not significant.

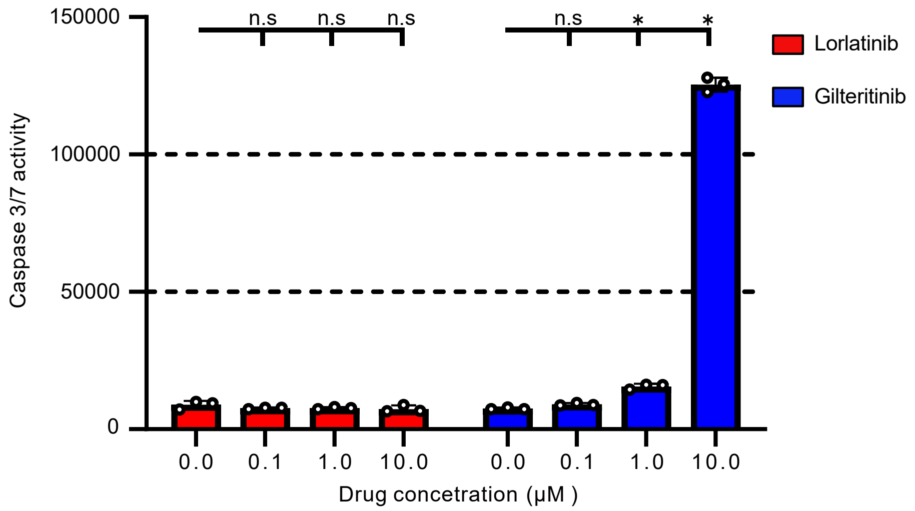

Supplementary Figure.3 Increase in caspase 3/7 activity in Ba/F3 cells expressing *CLIP1-LTK*-L650F treated with lorlatinib and gilteritinib at the indicated concentration for 8h. Caspase 3/7 activity was evaluated using the Caspase-Glo3/7 Assay system. Error bars are presented as mean± SD. * p<0.001 (Tukey's test); n.s, not significant.

Supplementary Figure.4 Uncropped scan images used in this manuscript. Cropped imaged used are indicated by red squares.

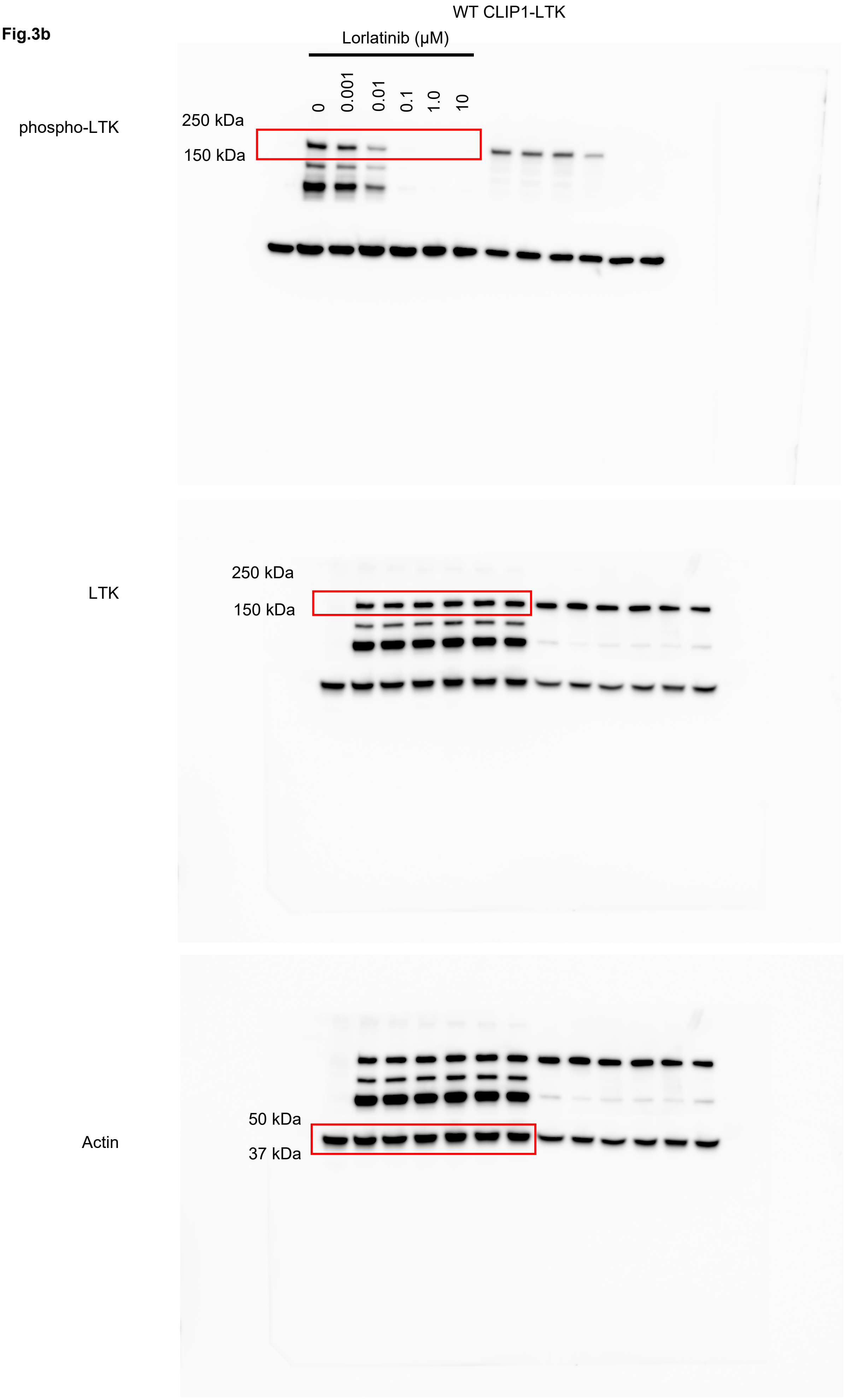

Fig.3b

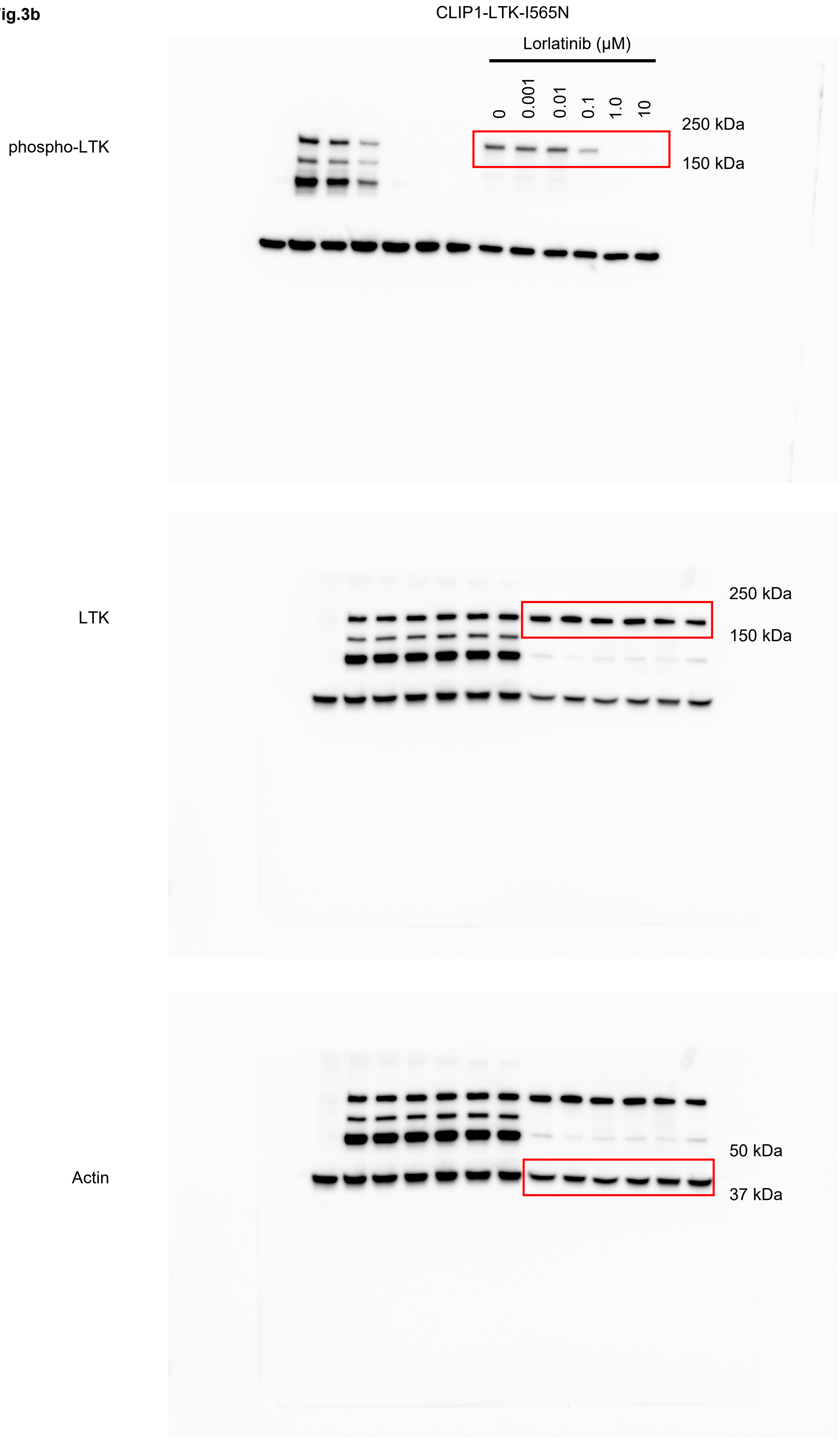
CLIP1-LTK-I565N
Lorlatinib (μM)
0
0.001
0.01
0.1
1.0
10
phospho-LTK
250 kDa
150 kDa
LTK
250 kDa
150 kDa
Actin
50 kDa
37 kDa

Fig.3b

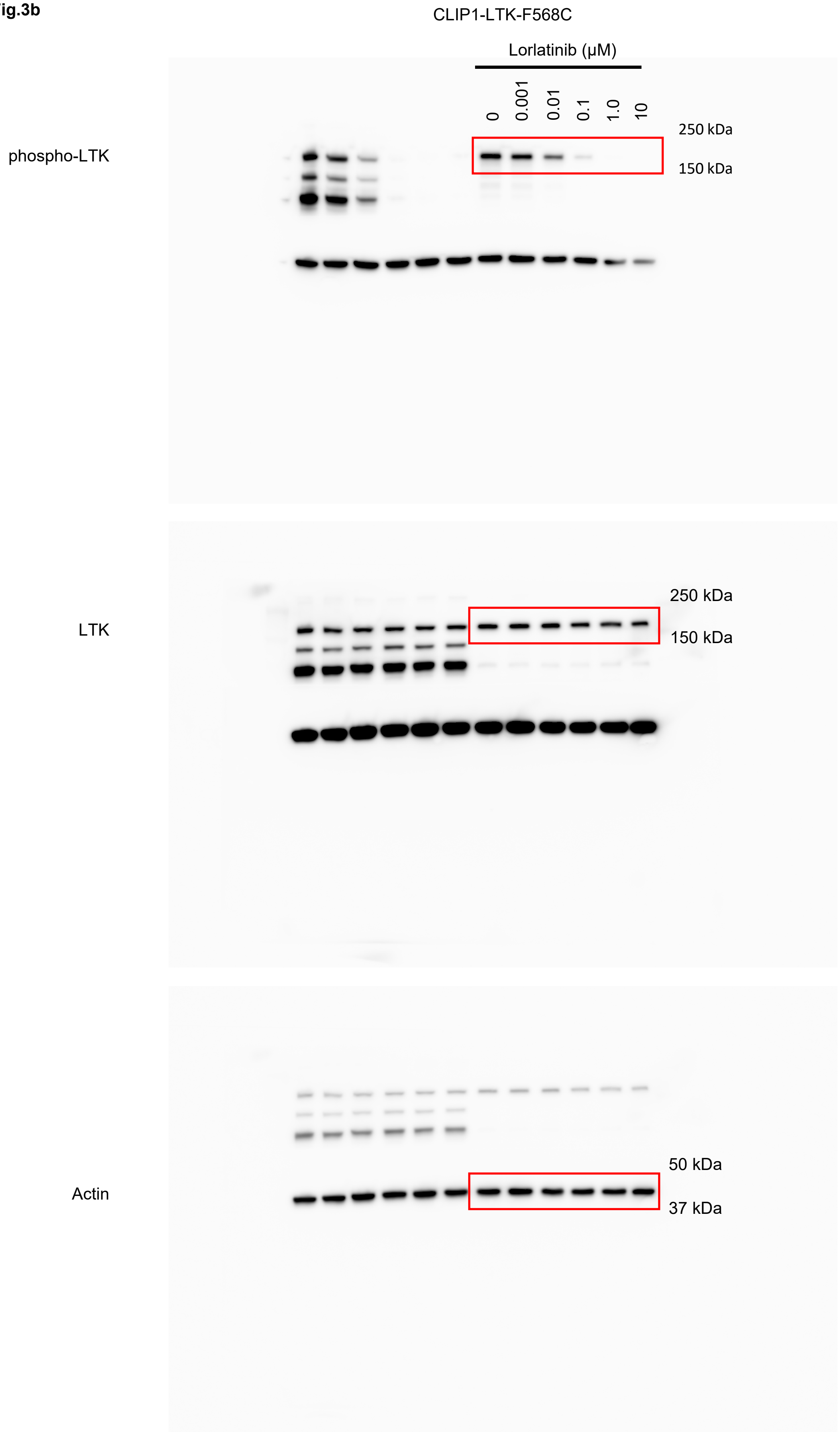
CLIP1-LTK-F568C
Lorlatinib (µM)
0
0.001
0.01
0.1
1.0
10
phospho-LTK
250 kDa
150 kDa
LTK
250 kDa
150 kDa
Actin
50 kDa
37 kDa

Fig.3b

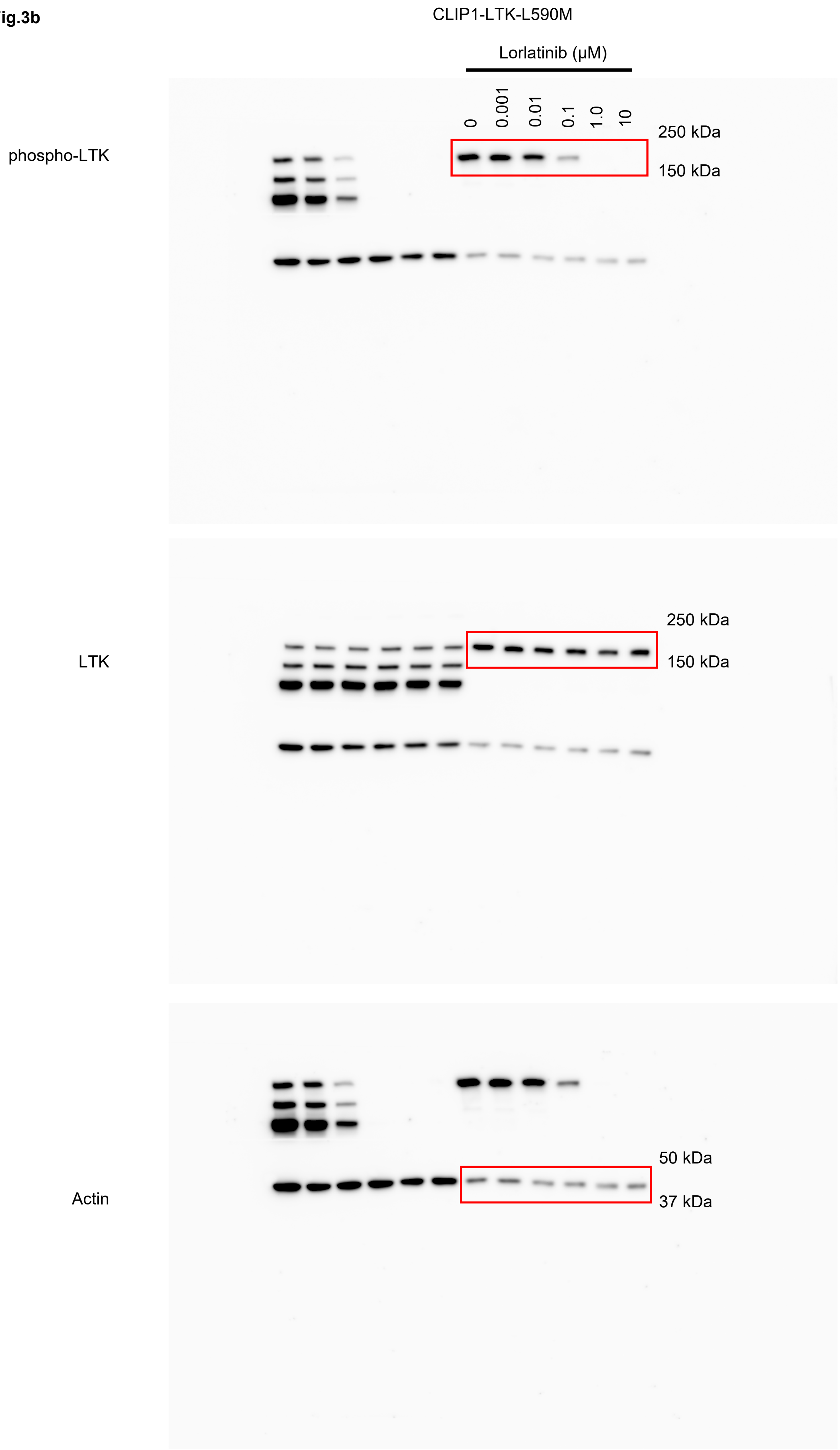

CLIP1-LTK-L590M
Lorlatinib (μM)
0
0.001
0.01
0.1
1.0
10
phospho-LTK
250 kDa
150 kDa
LTK
250 kDa
150 kDa
Actin
50 kDa
37 kDa

Fig.3b

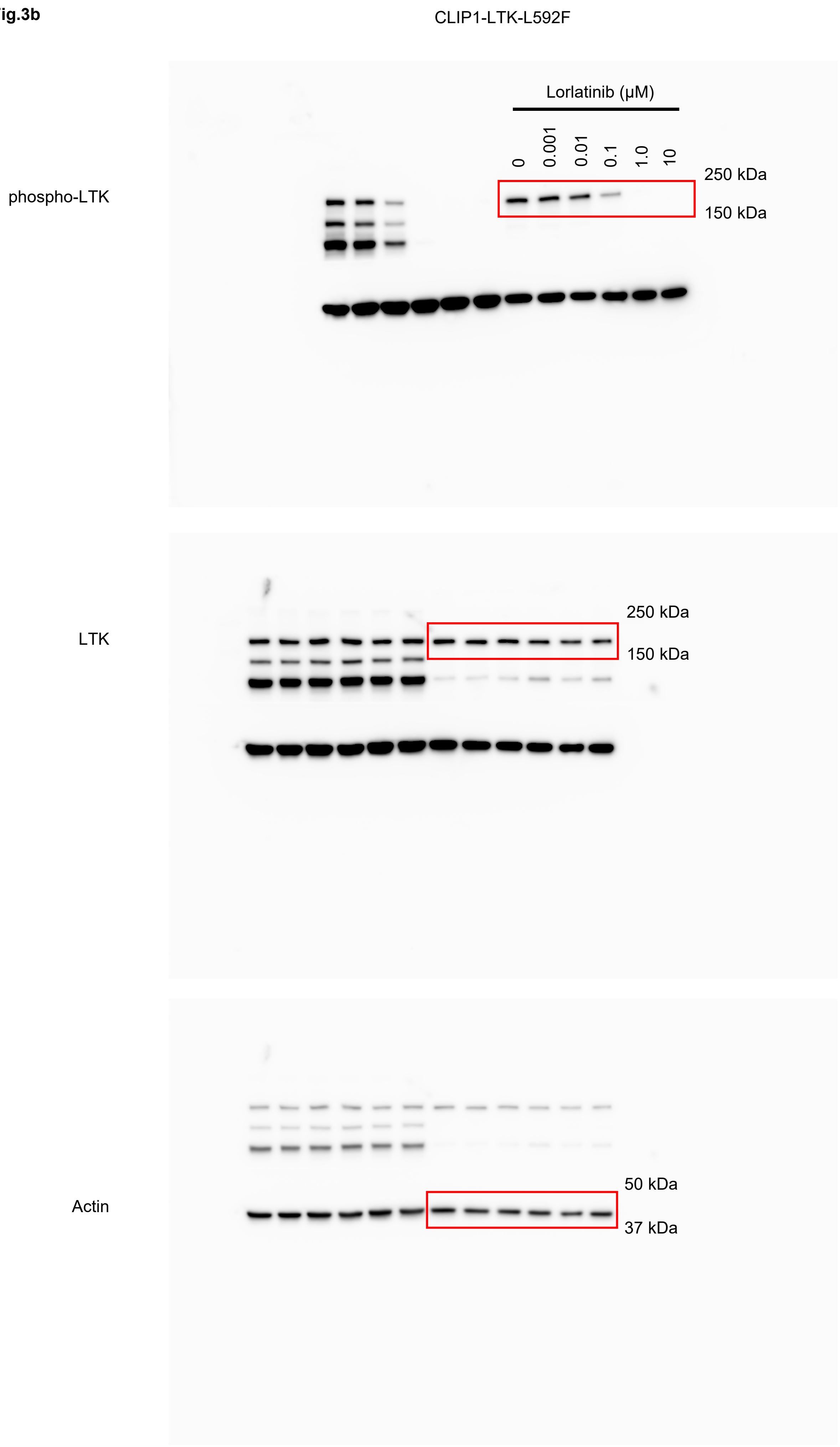
CLIP1-LTK-L592F
phospho-LTK
Lorlatinib (μM)
0
0.001
0.01
0.1
1.0
10
250 kDa
150 kDa
LTK
250 kDa
150 kDa
Actin
50 kDa
37 kDa

Fig.3b

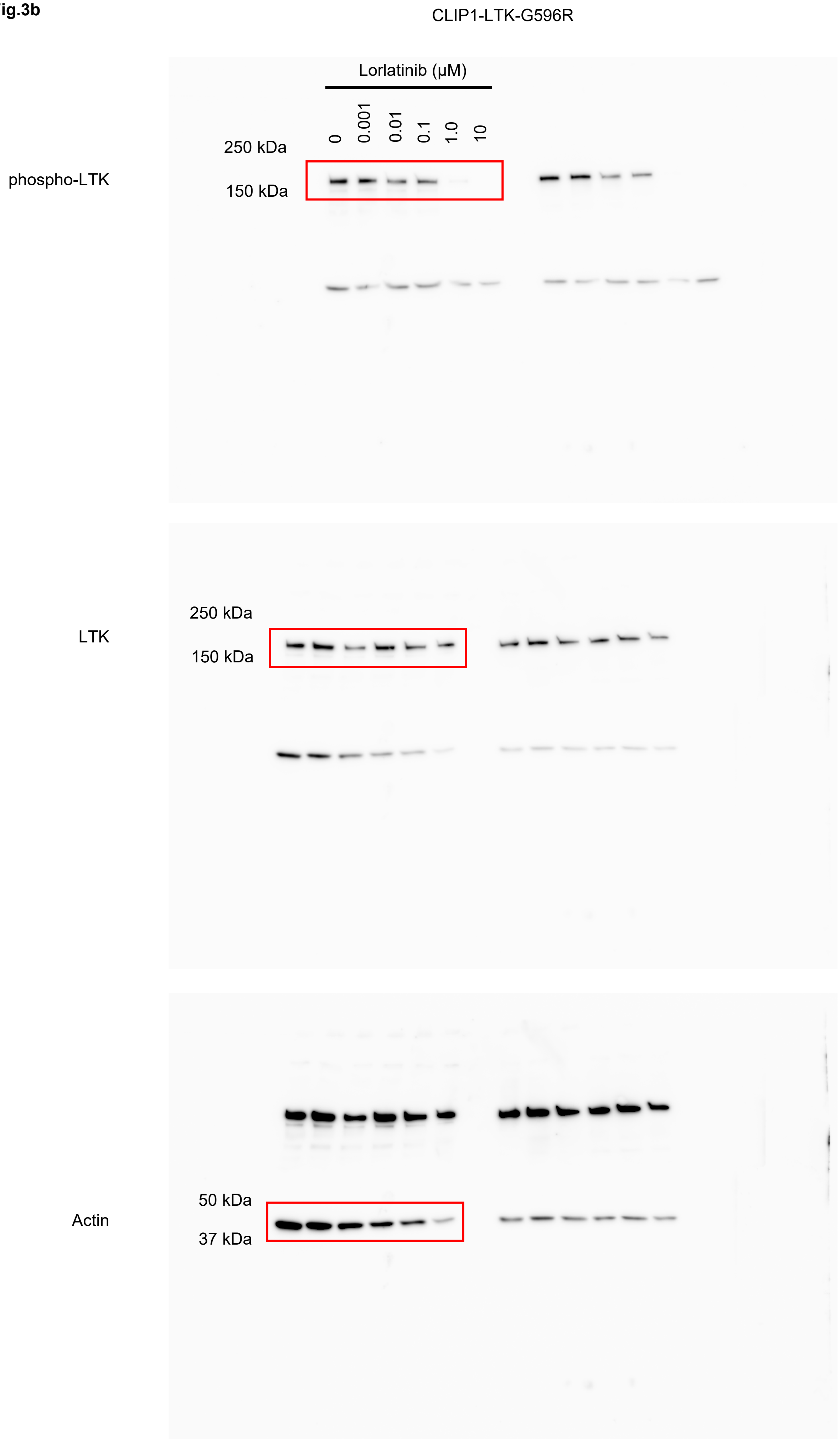
CLIP1-LTK-G596R
Lorlatinib (μM)
0
0.001
0.01
0.1
1.0
10
phospho-LTK
250 kDa
150 kDa
LTK
250 kDa
150 kDa
Actin
50 kDa
37 kDa

Fig.3b

CLIP1-LTK-D597N

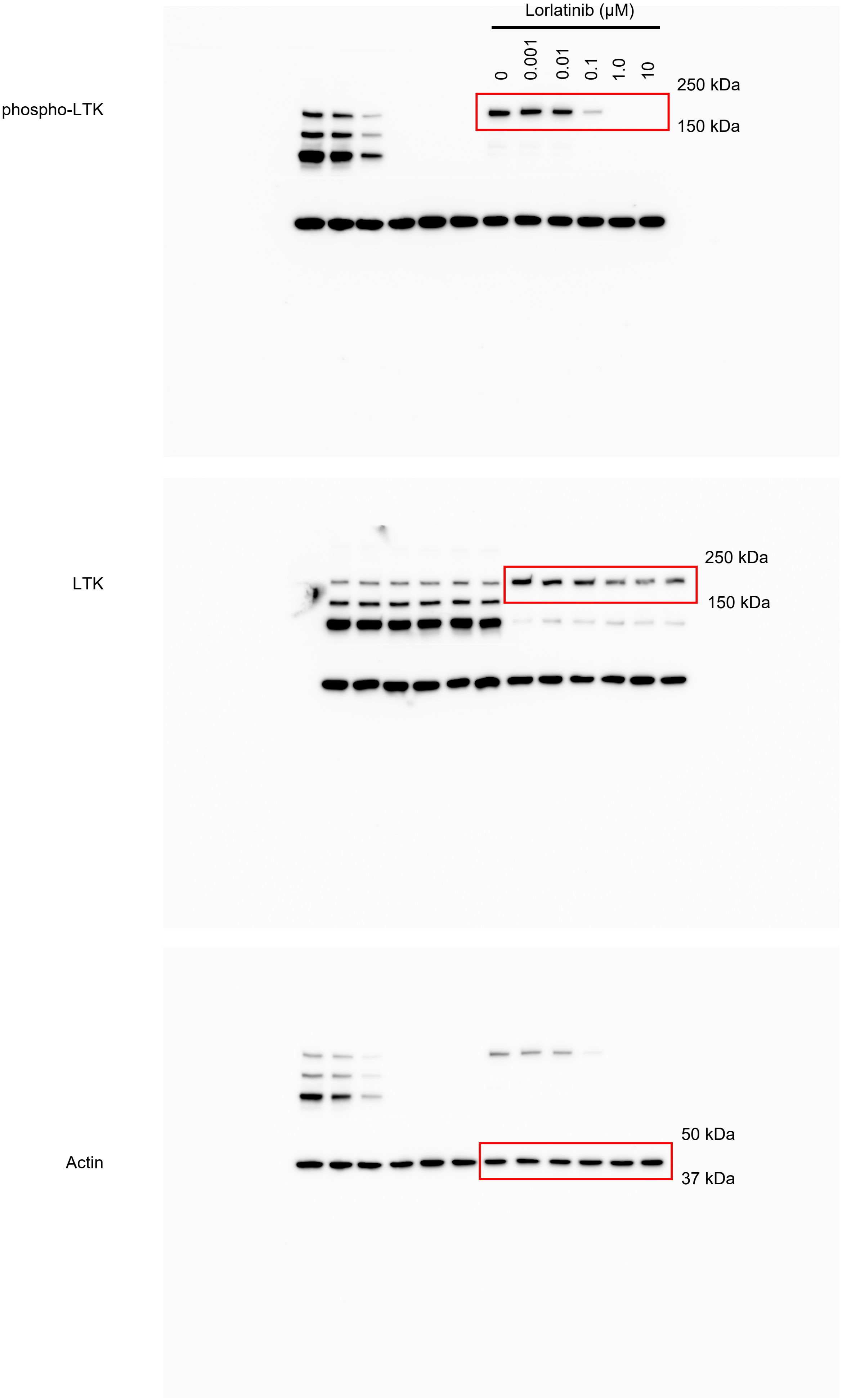

Fig.3b

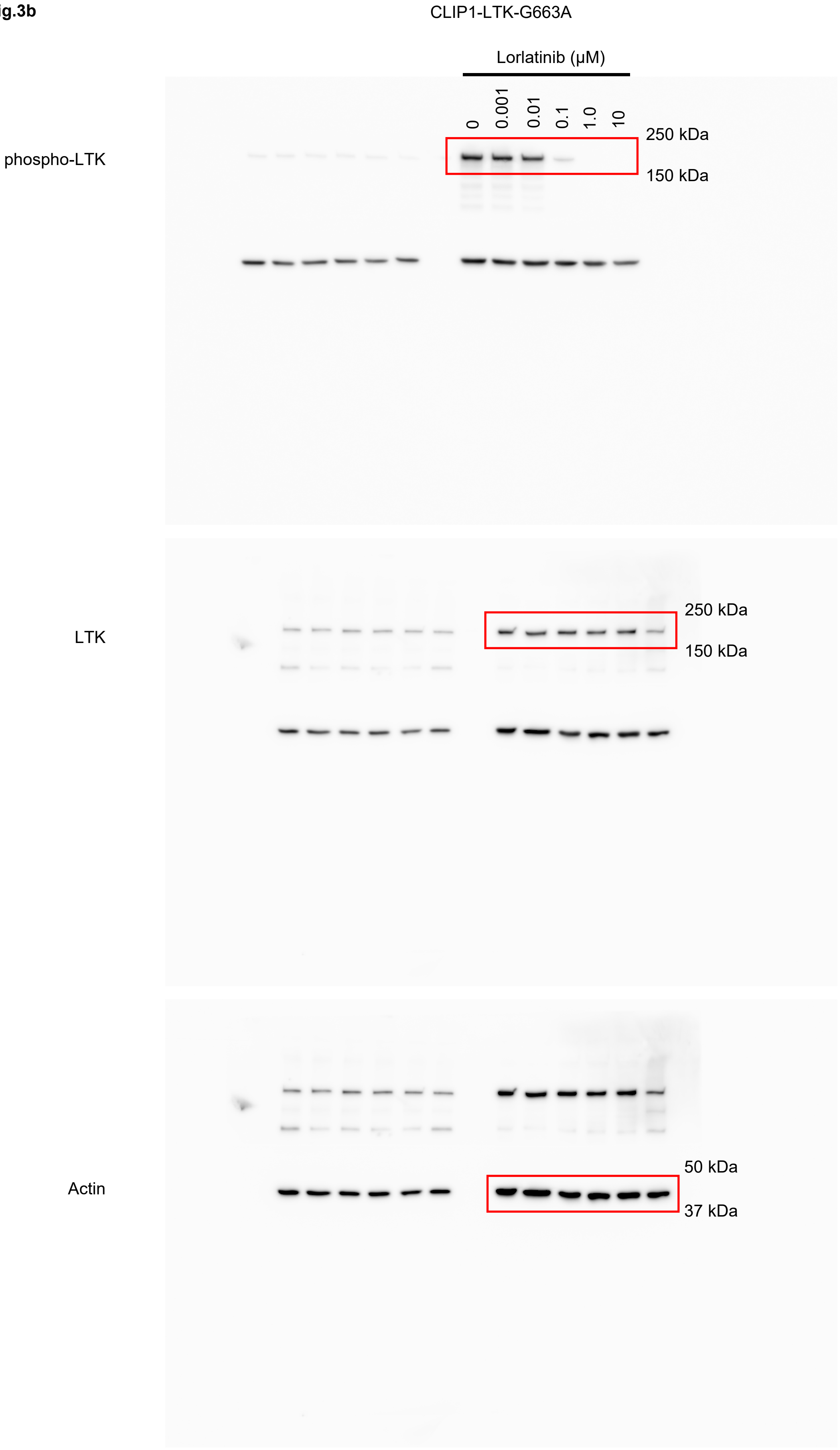

CLIP1-LTK-G663A
Lorlatinib (μM)
0
0.001
0.01
0.1
1.0
10
phospho-LTK
250 kDa
150 kDa
LTK
250 kDa
150 kDa
Actin
50 kDa
37 kDa

Fig.3b

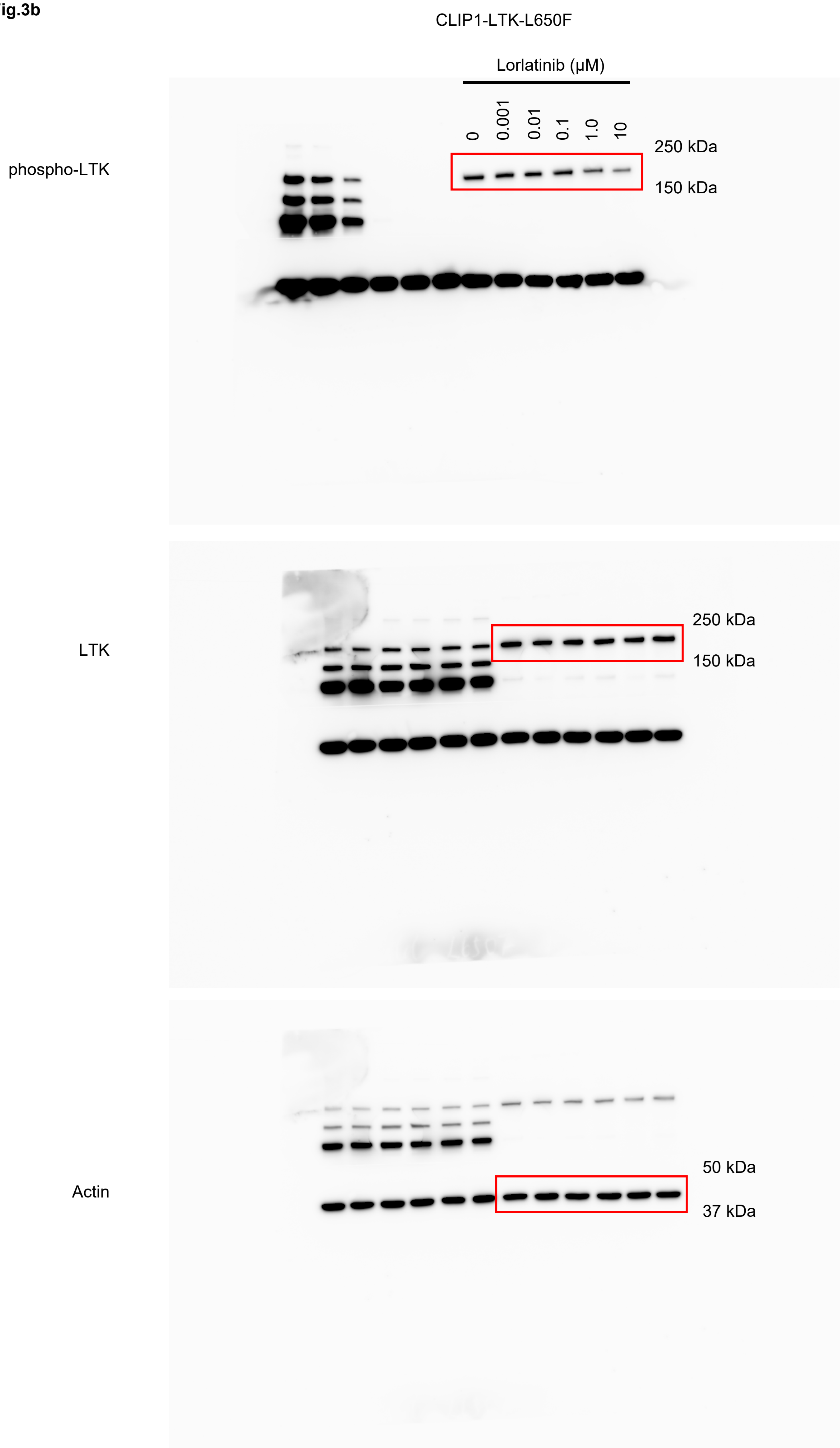
CLIP1-LTK-L650F
Lorlatinib (μM)
0
0.001
0.01
0.1
1.0
10
phospho-LTK
250 kDa
150 kDa
LTK
250 kDa
150 kDa
Actin
50 kDa
37 kDa

Fig.5b

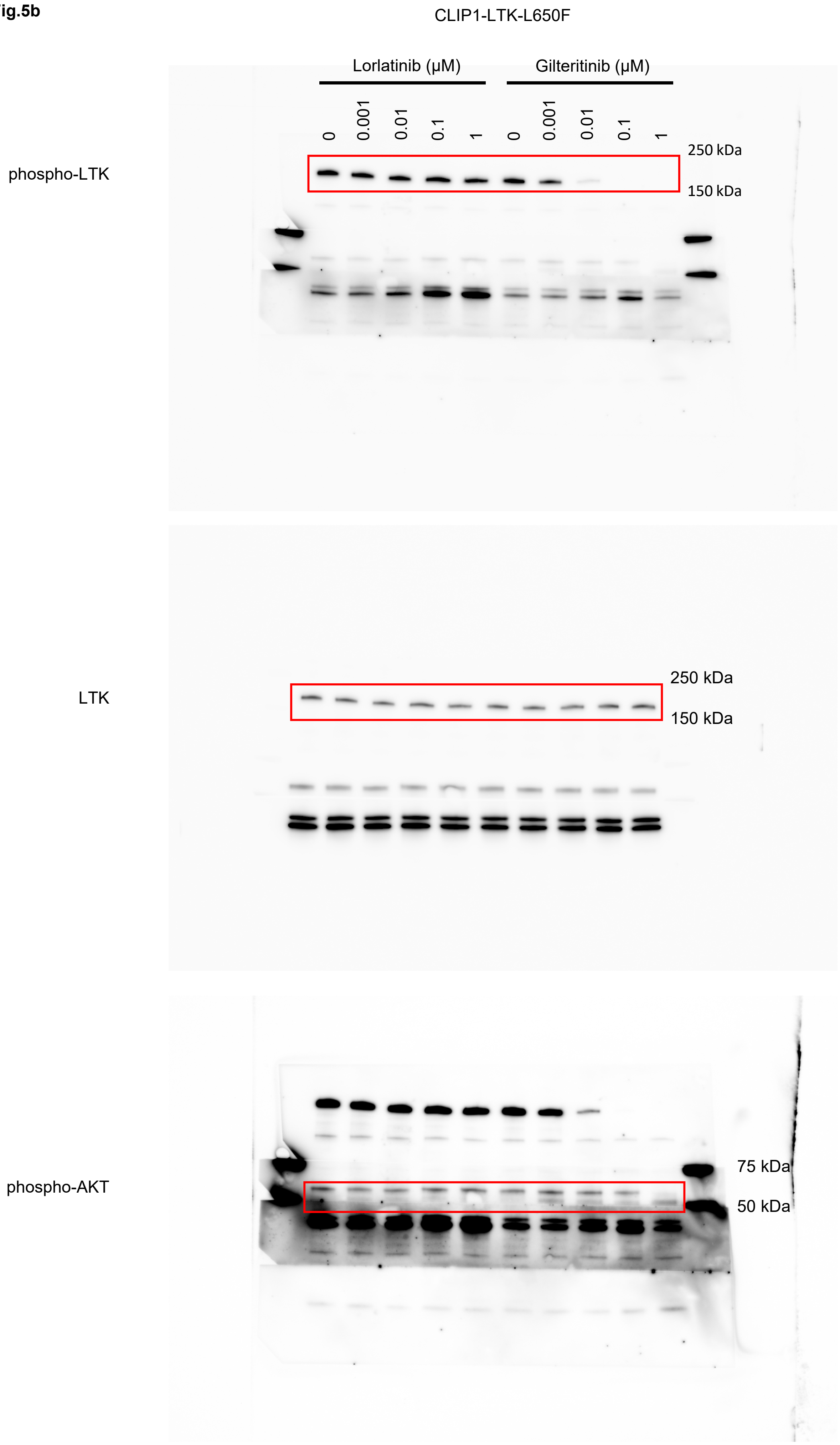
CLIP1-LTK-L650F
Lorlatinib (μM)
Gilteritinib (μM)
0
0.001
0.01
0.1
1
0
0.001
0.01
0.1
1
phospho-LTK
250 kDa
150 kDa
LTK
250 kDa
150 kDa
phospho-AKT
75 kDa
50 kDa

Fig.5b

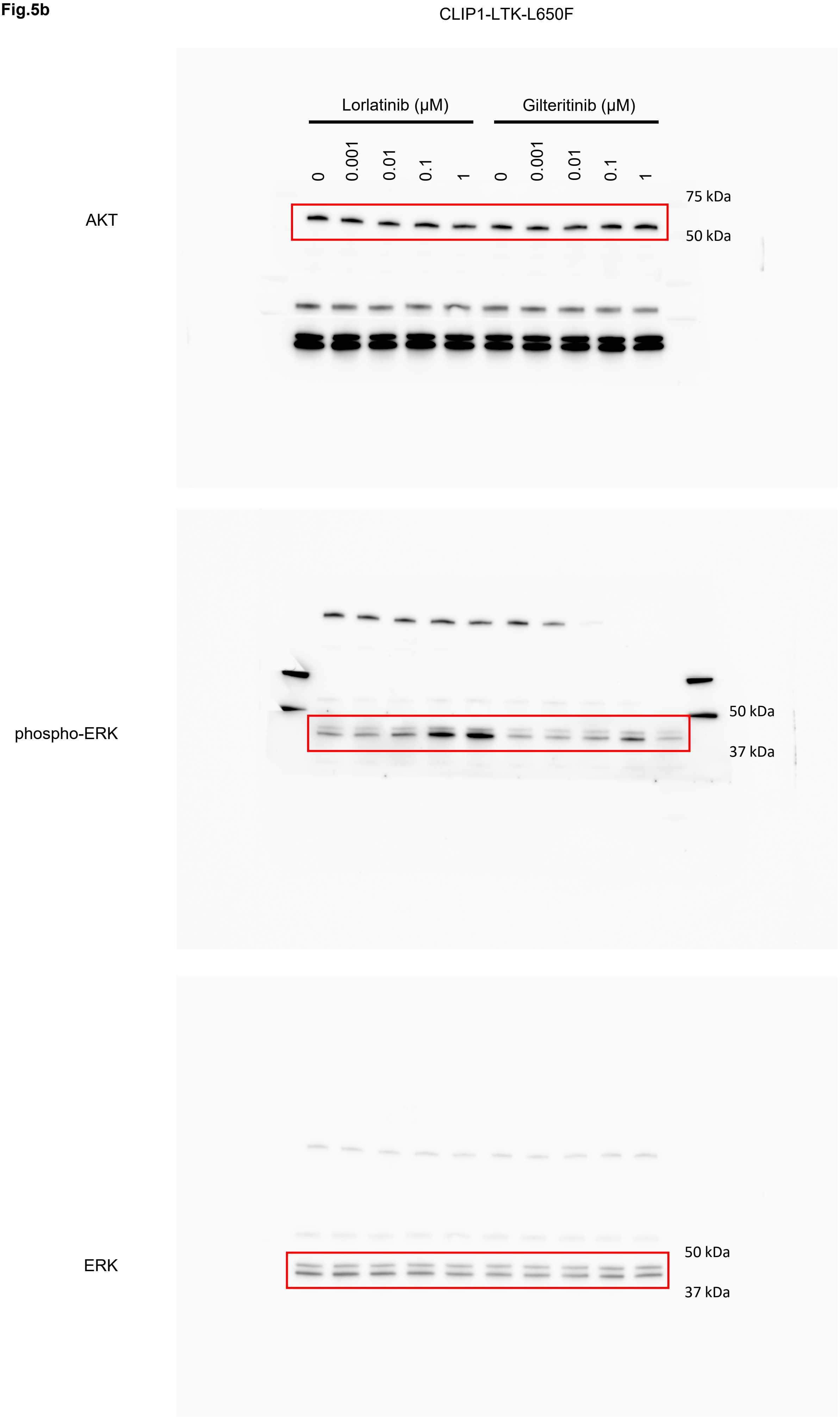
CLIP1-LTK-L650F
Lorlatinib (μM)
Gilteritinib (μM)
0
0.001
0.01
0.1
1
0
0.001
0.01
0.1
1
AKT
75 kDa
50 kDa
phospho-ERK
50 kDa
37 kDa
ERK
50 kDa
37 kDa

Fig.5b

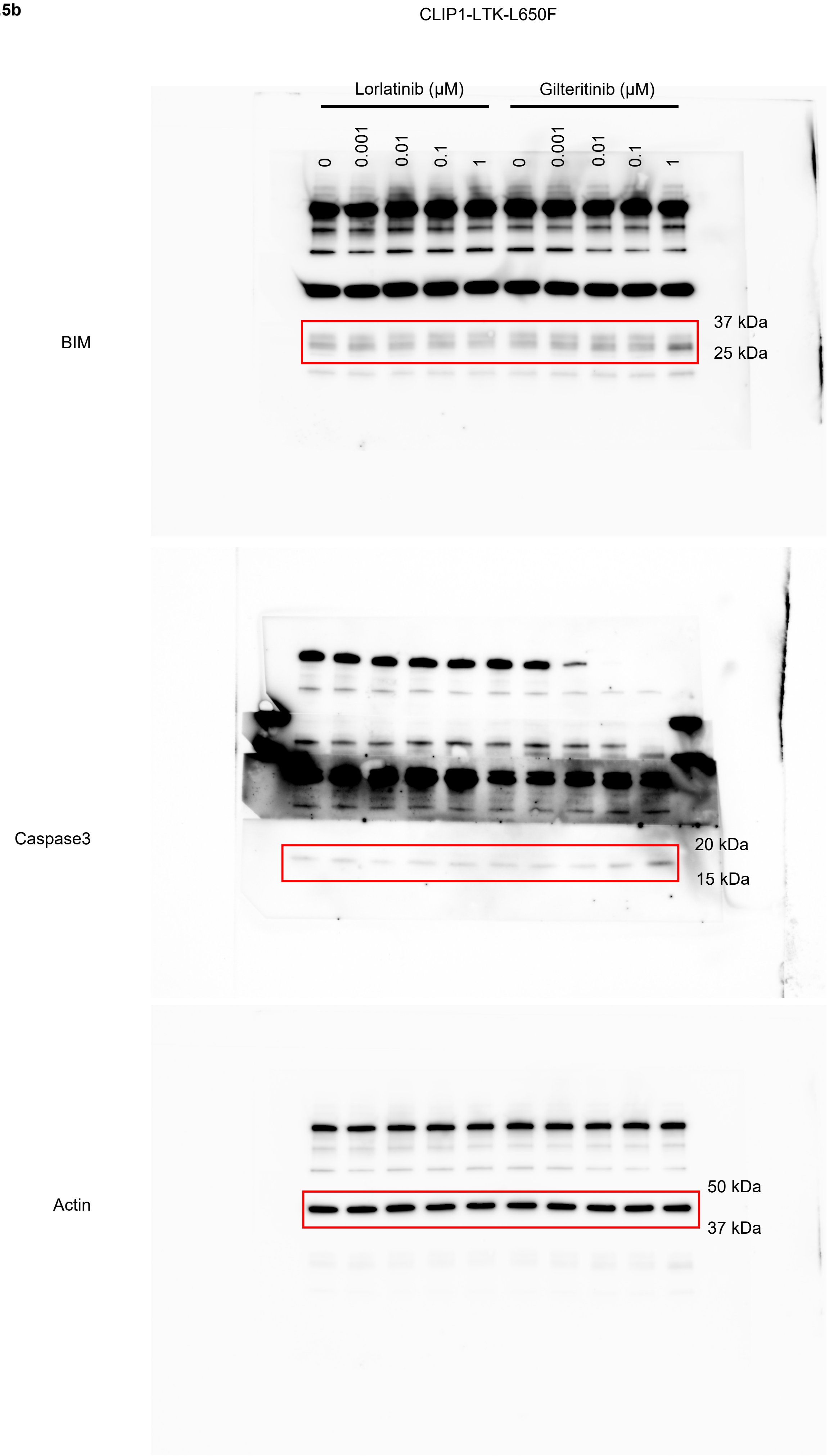
CLIP1-LTK-L650F
Lorlatinib (μM)
Gilteritinib (μM)
0
0.001
0.01
0.1
1
0
0.001
0.01
0.1
1
BIM
37 kDa
25 kDa
Caspase3
20 kDa
15 kDa
Actin
50 kDa
37 kDa

Supplementary Fig.1

CLIP1-LTK-G596R

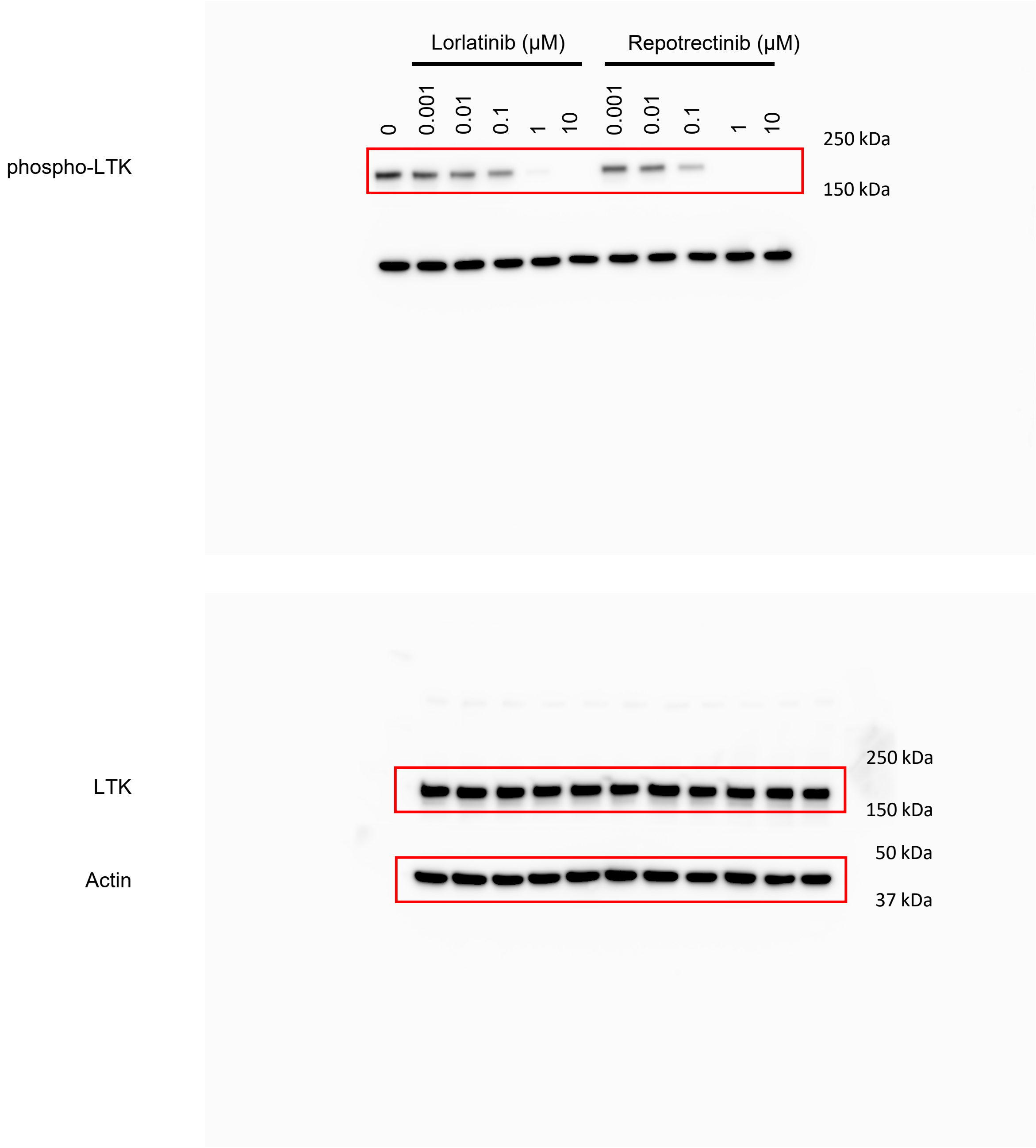

Supplementary Fig.1

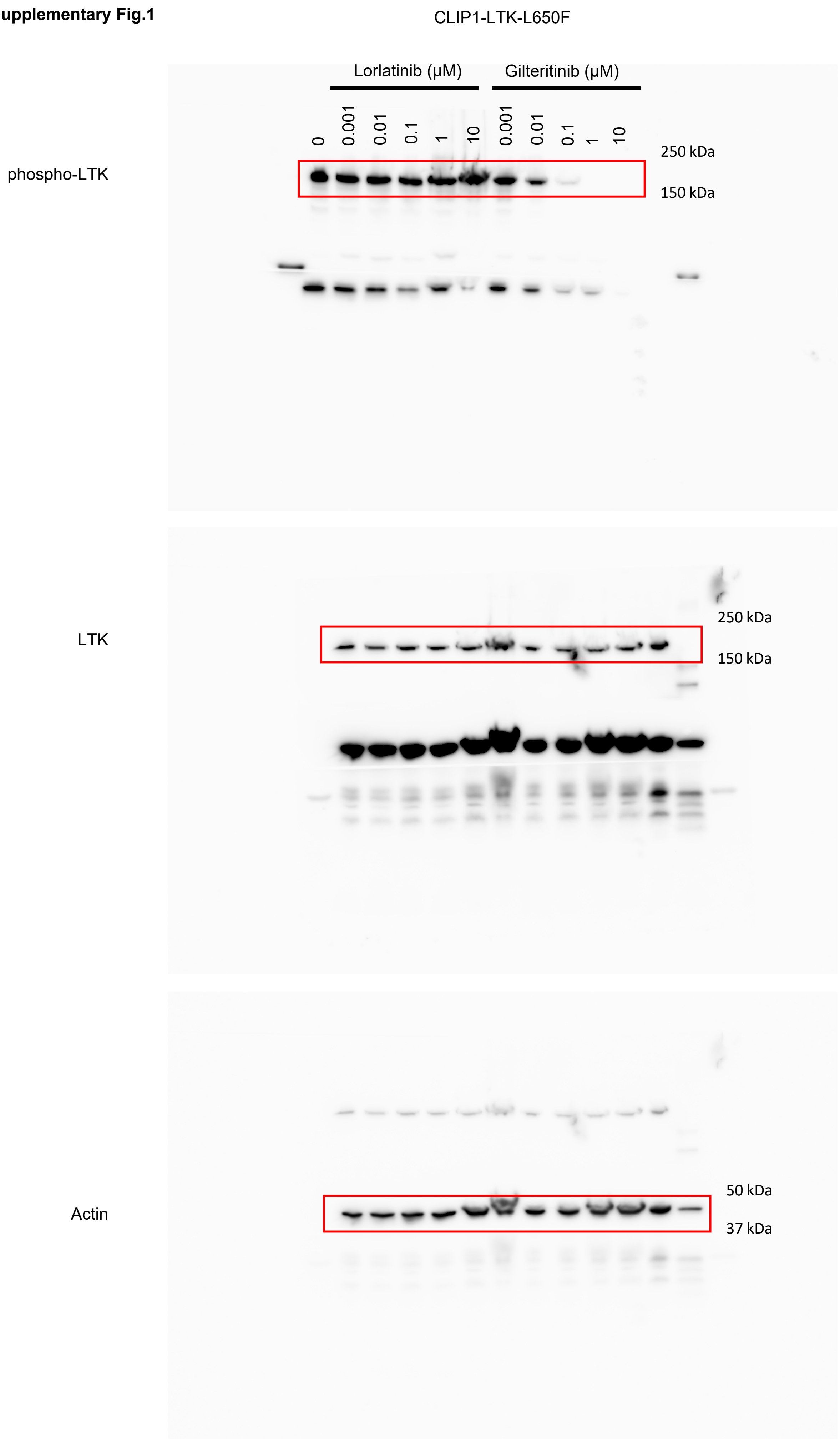

Supplementary Fig.1

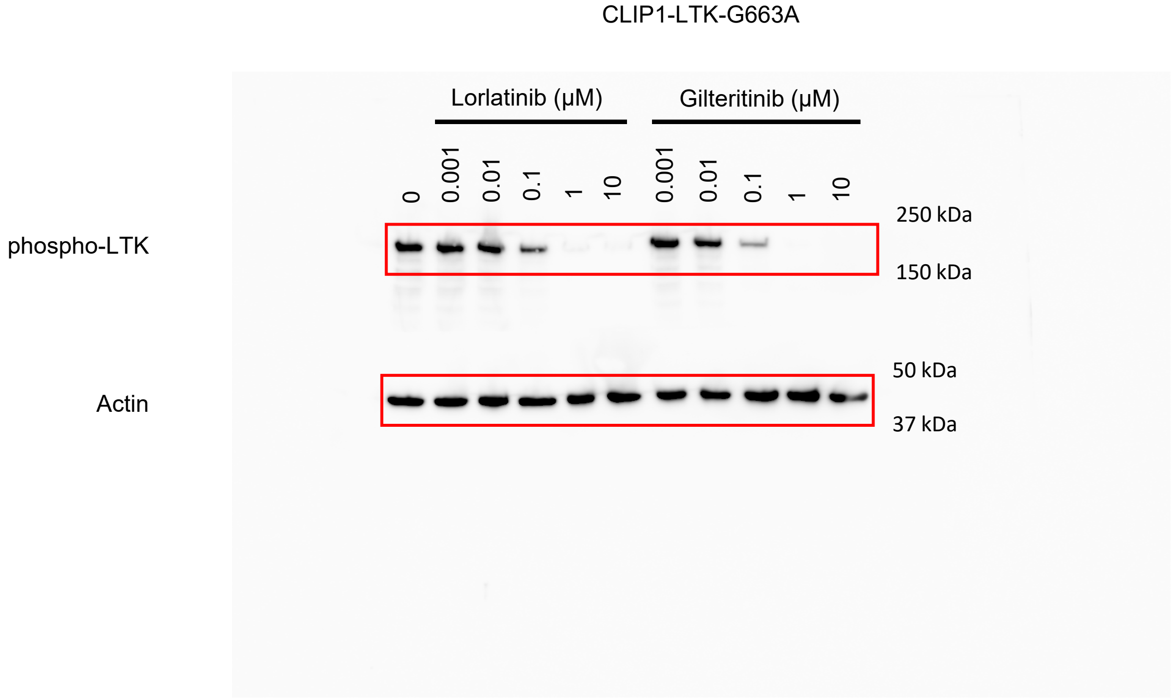

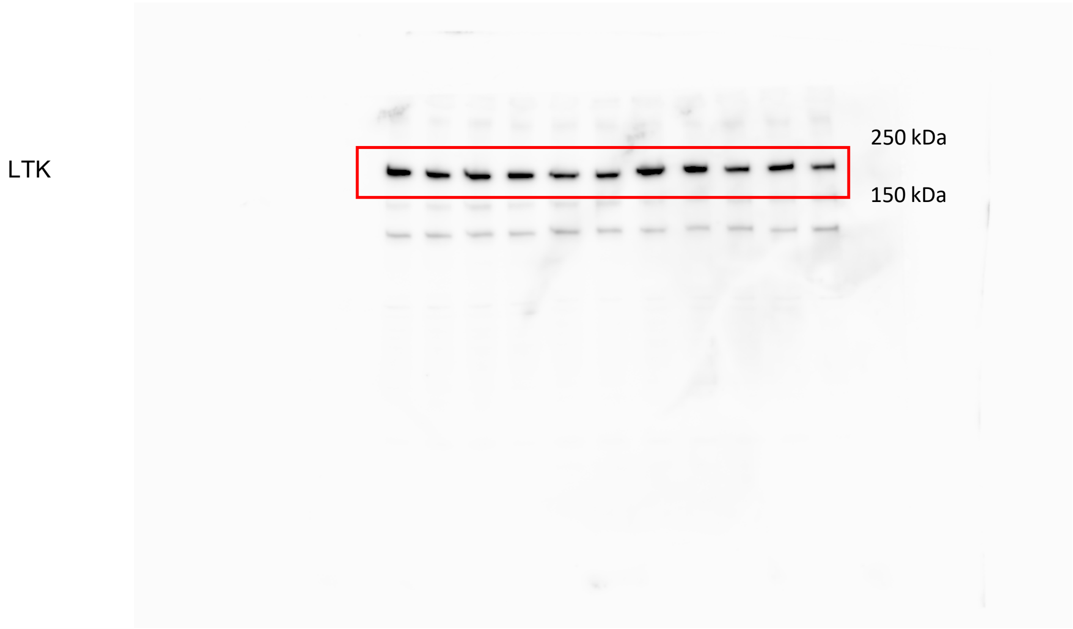

Supplementary Figure.5 Gating strategy for live cells. Total cells are gated on FSC-H/FSC-A plot where viable cells are selected as single cells, and then analyzed as shown Fig. 3c and Fig. 5c. PI, Propidum iodide

Fig.3c

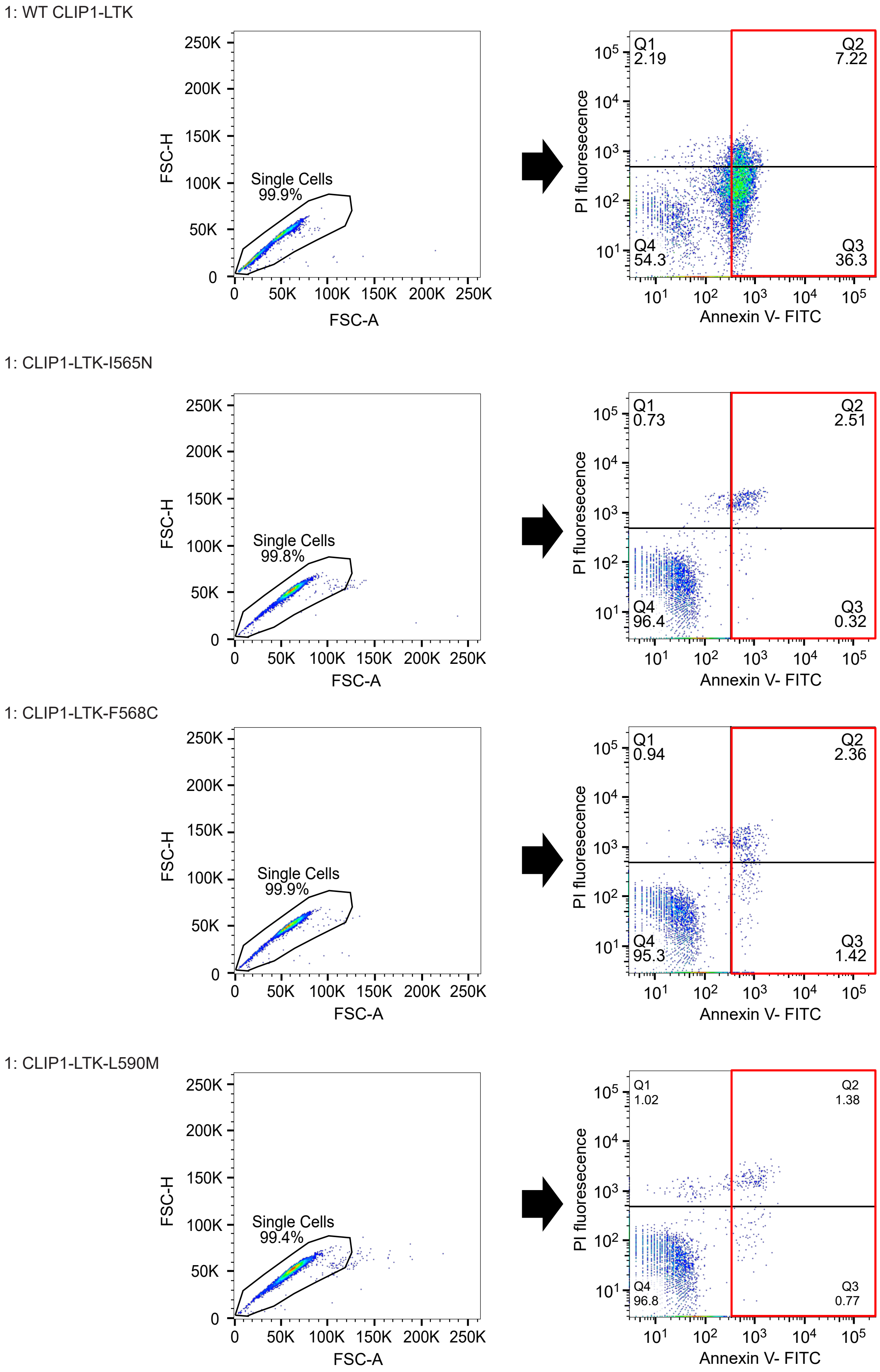

Fig.3c

1: CLIP1-LTK-L592F

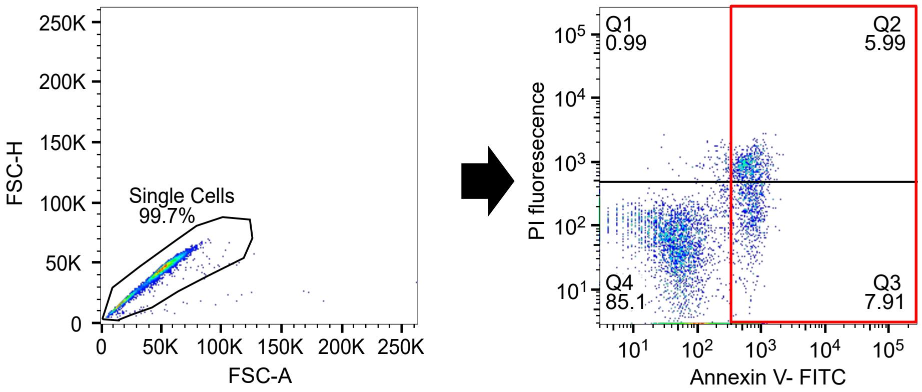

1: CLIP1-LTK-G596R

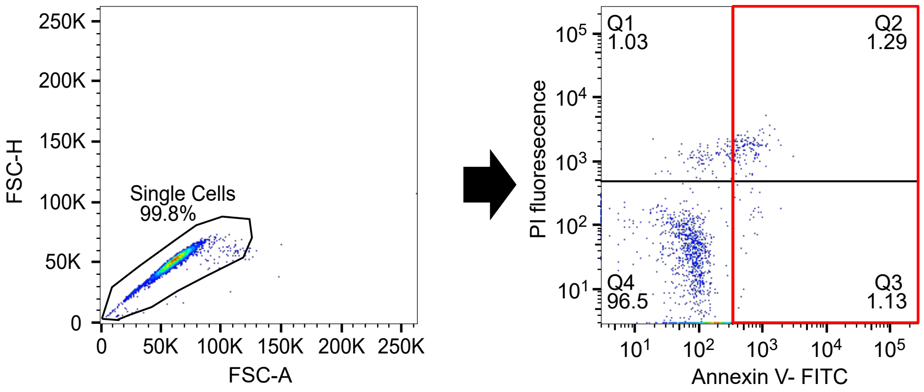

1: CLIP1-LTK-D597N

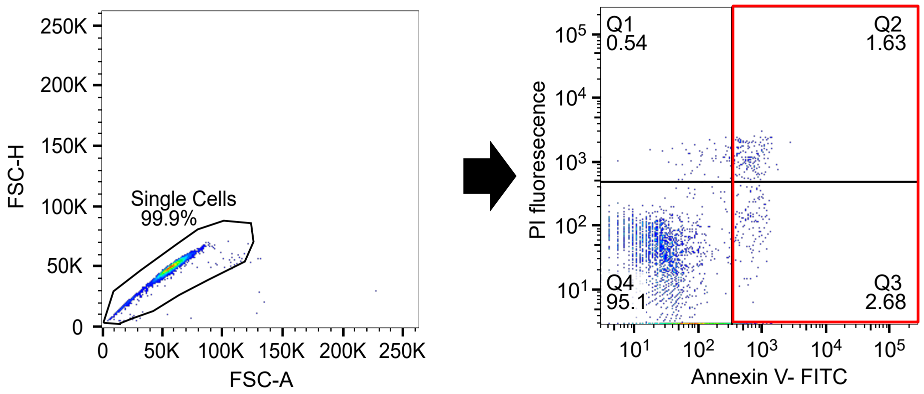

1: CLIP1-LTK-L650F

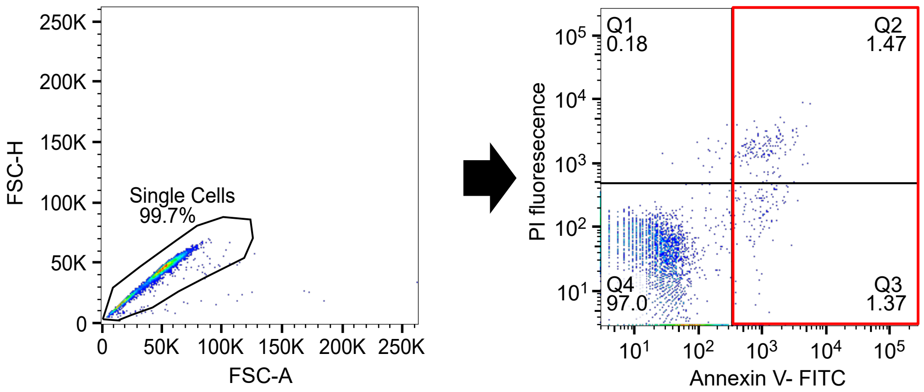

Fig.3c

1: CLIP1-LTK-G663A

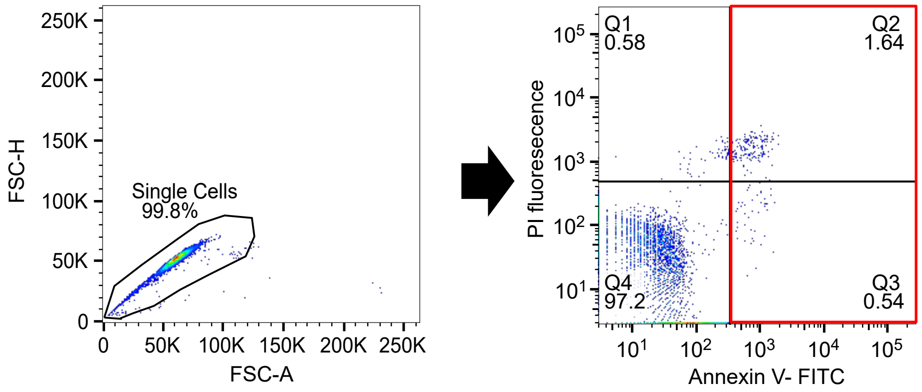

Fig.3c

2: WT CLIP1-LTK

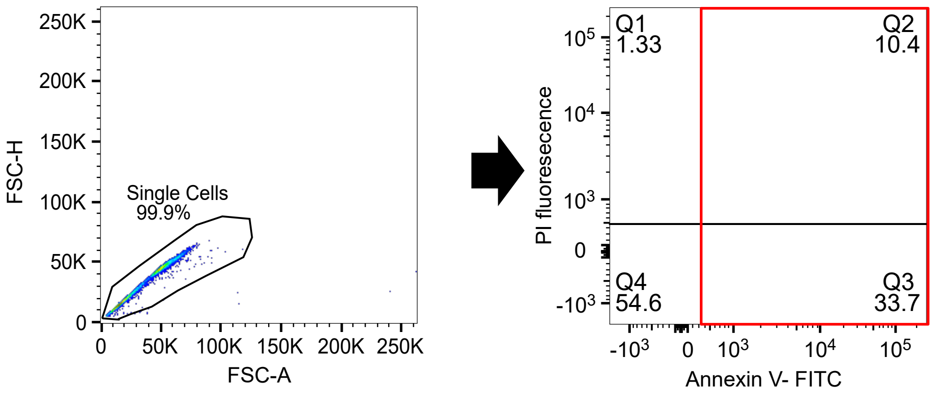

2: CLIP1-LTK-I565N

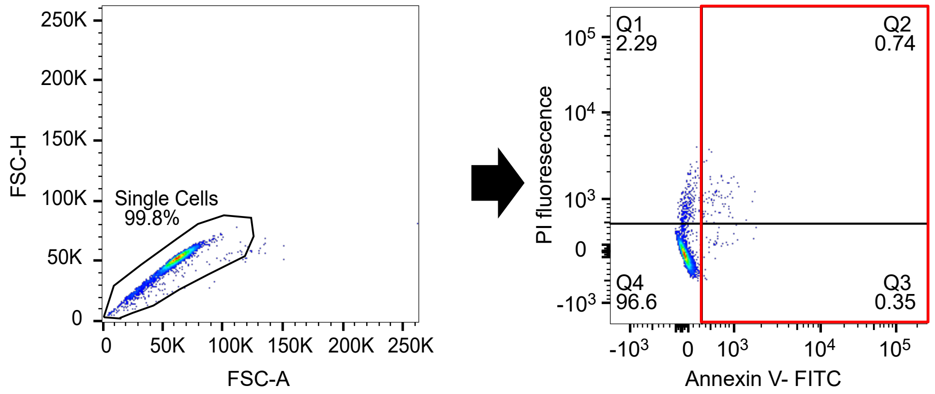

2: CLIP1-LTK-F568C

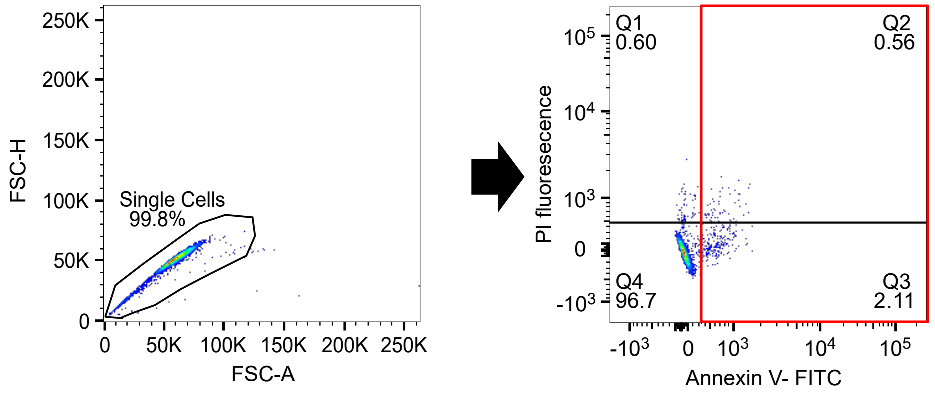

2: CLIP1-LTK-L590M

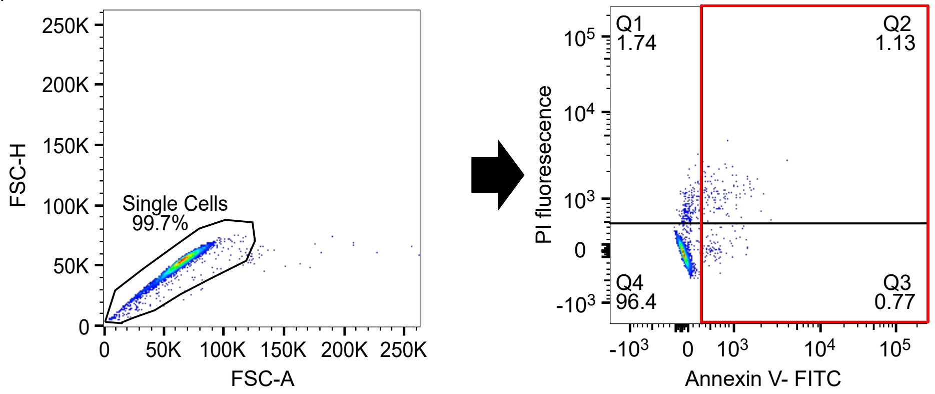

Fig.3c

2: CLIP1-LTK-L592F

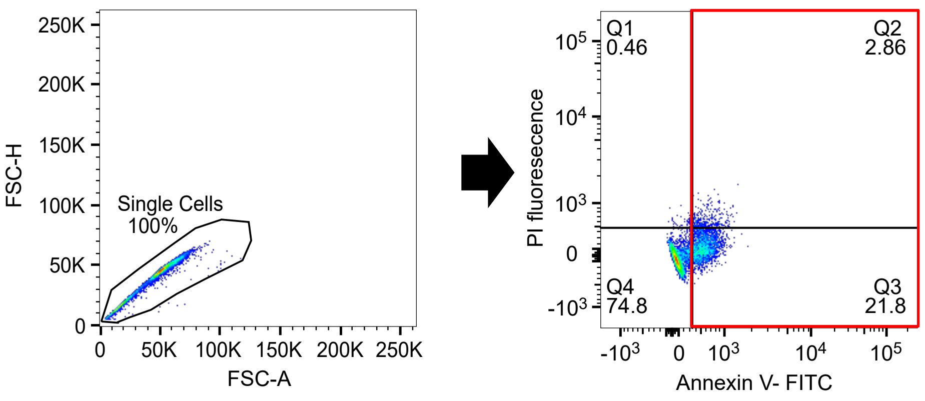

2: CLIP1-LTK-G596R

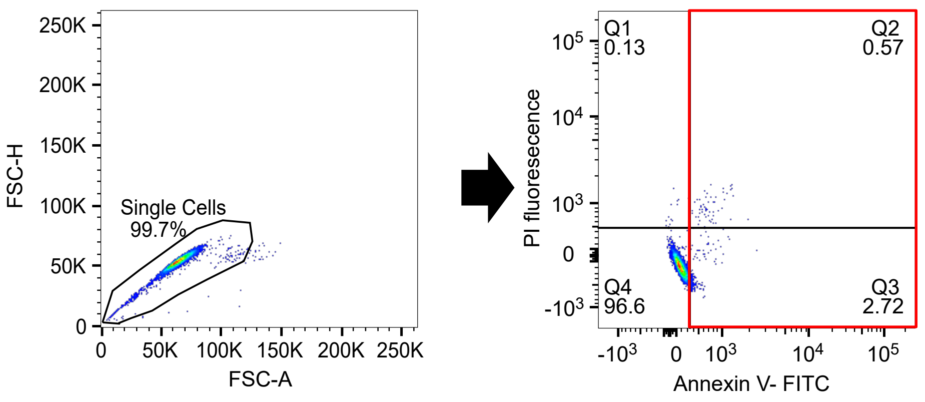

2: CLIP1-LTK-D597N

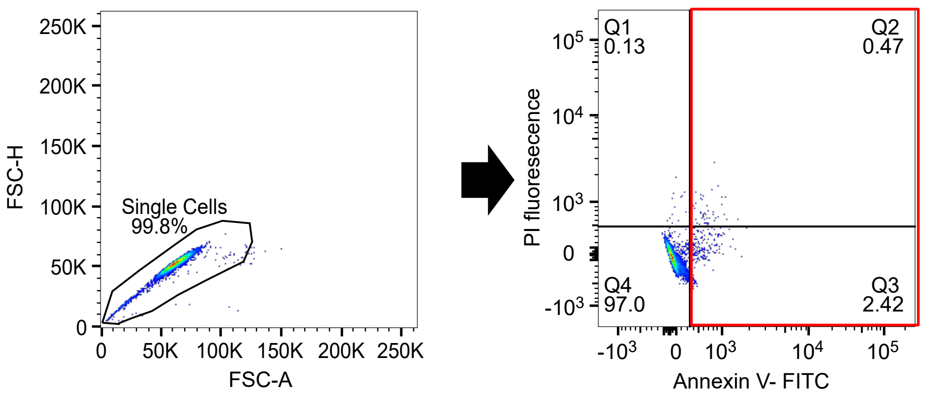

2: CLIP1-LTK-L650F

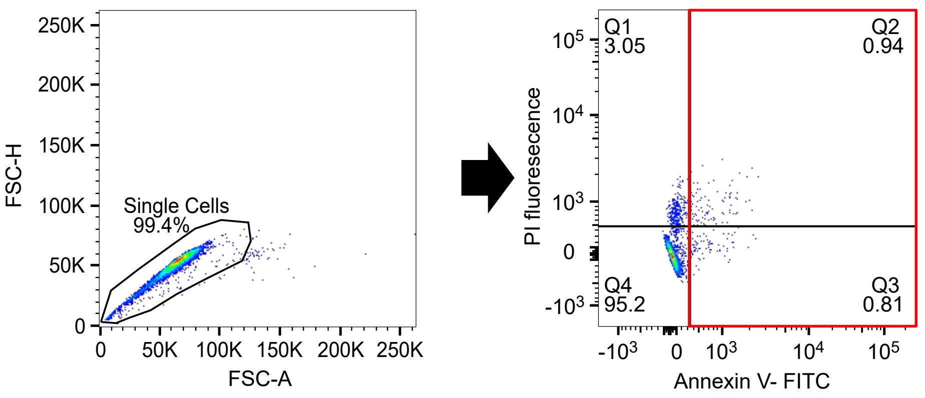

Fig.3c

2: CLIP1-LTK-G663A

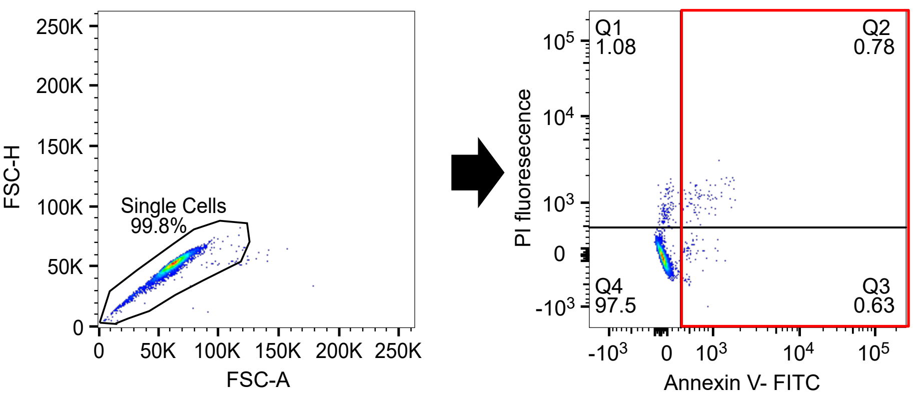

Fig.5c CLIP1-LTK-L650F

1: DMSO

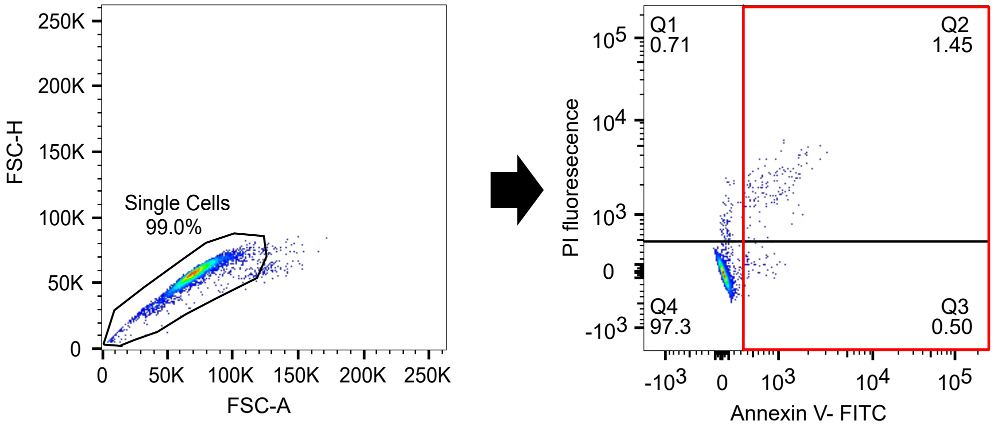

1: Lorlatinib

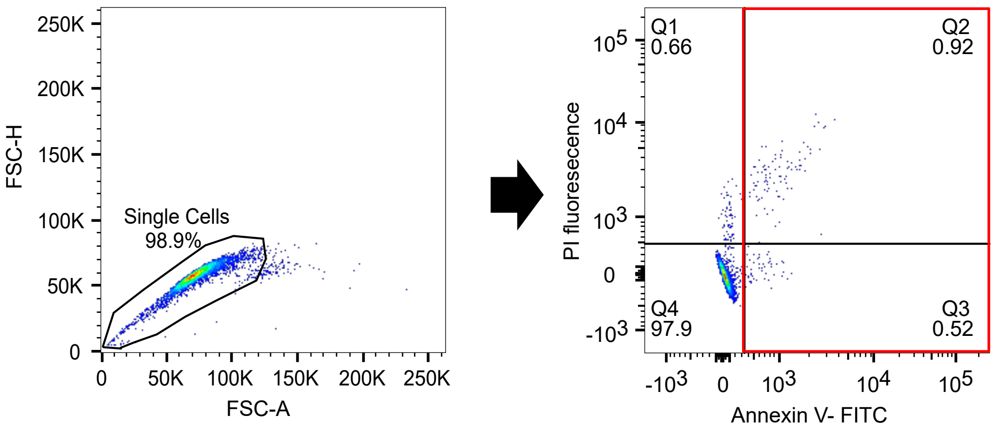

1: Gilteritinib

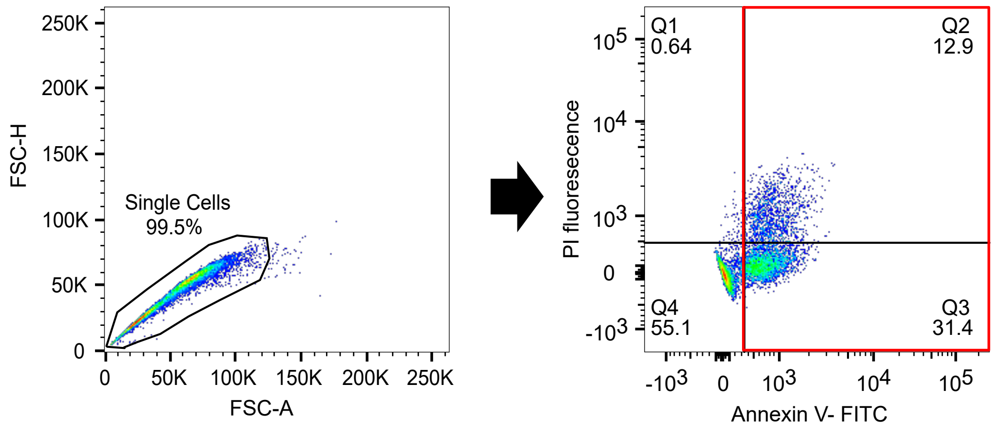

2: DMSO

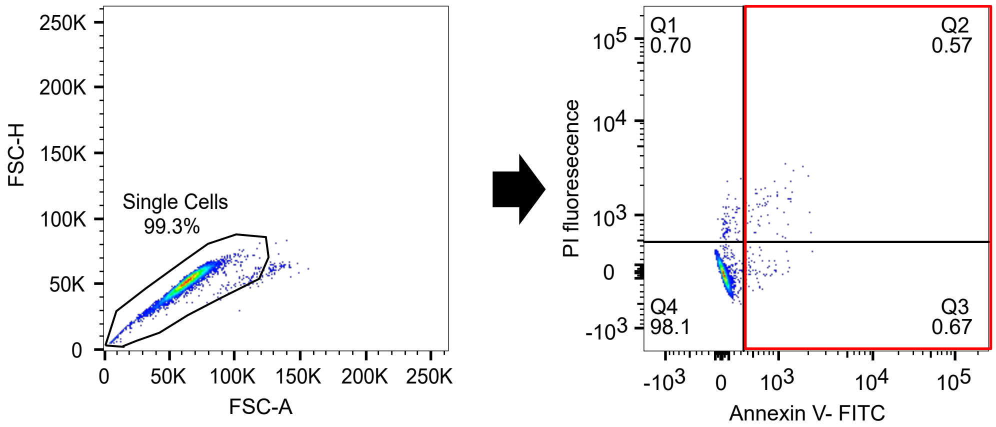

2: Lorlatinib

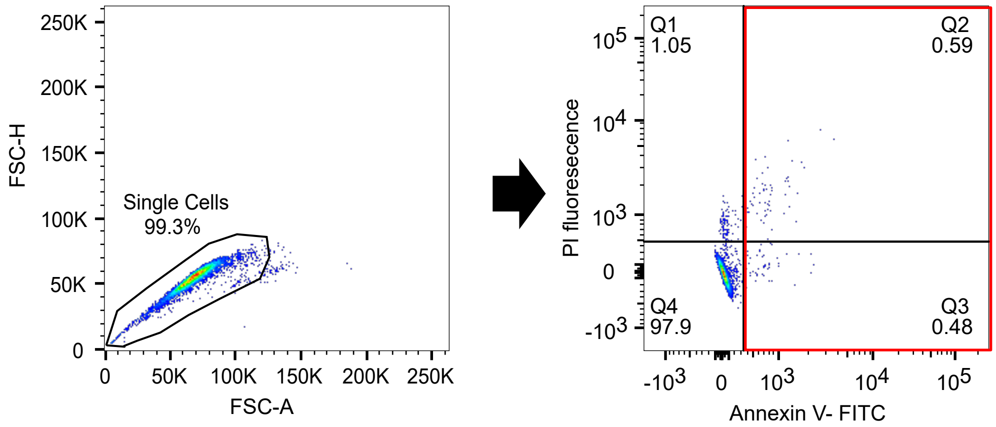

2: Gilteritinib

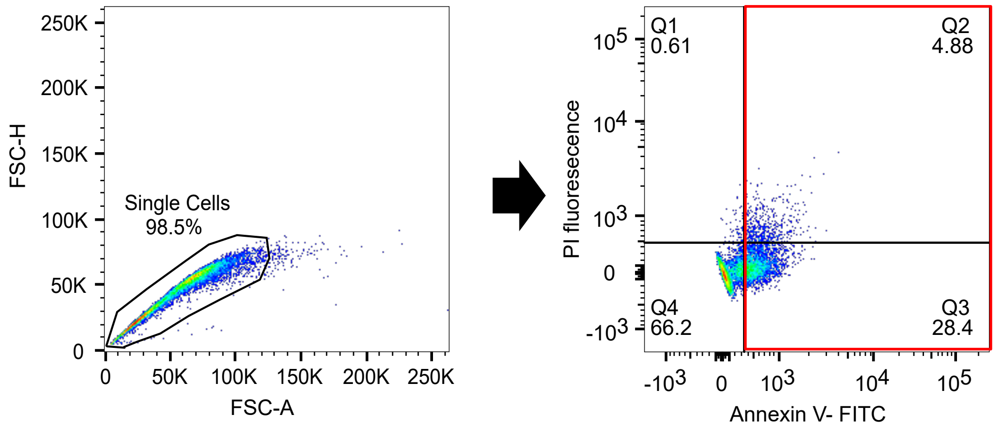

3: DMSO

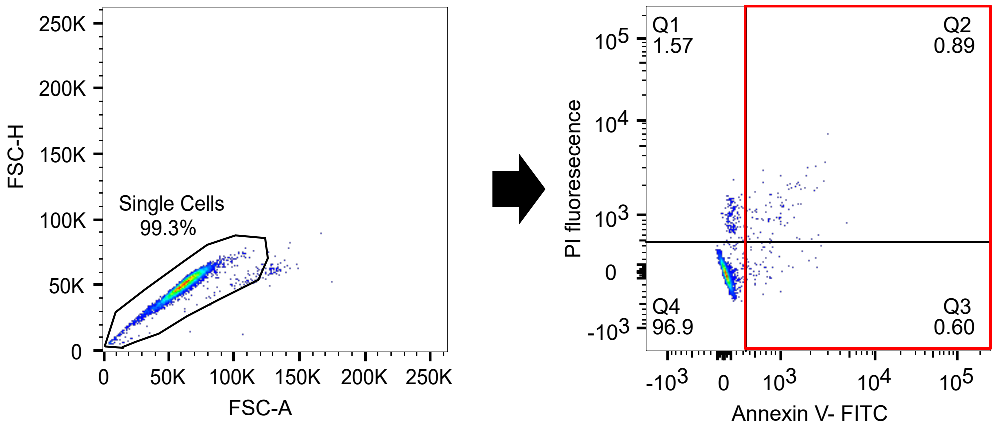

3: Lorlatinib

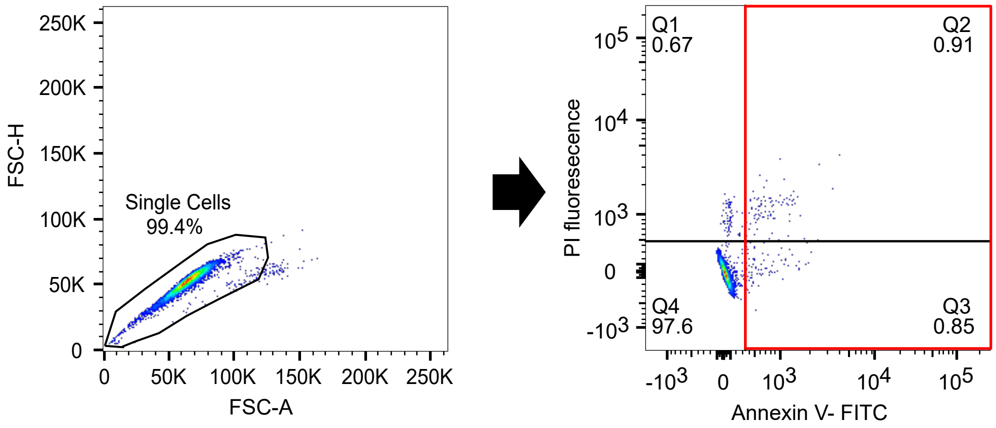

3: Gilteritinib

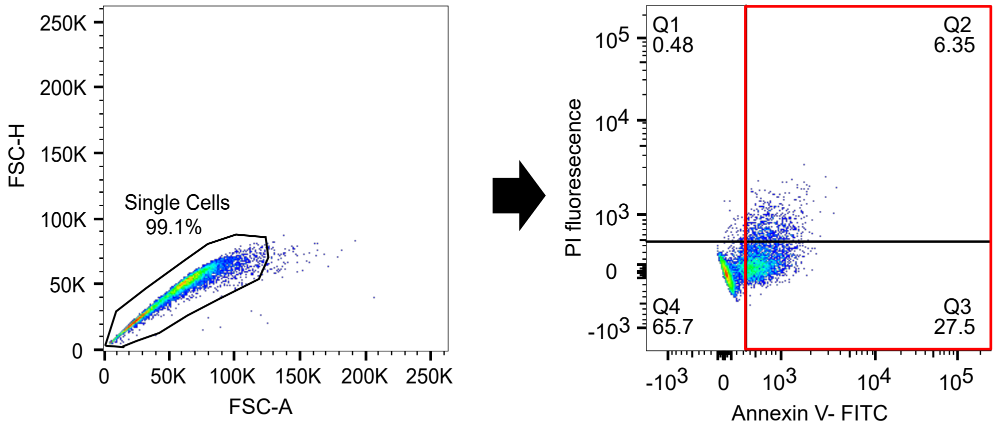

Fig.3c

3: WT CLIP1-LTK

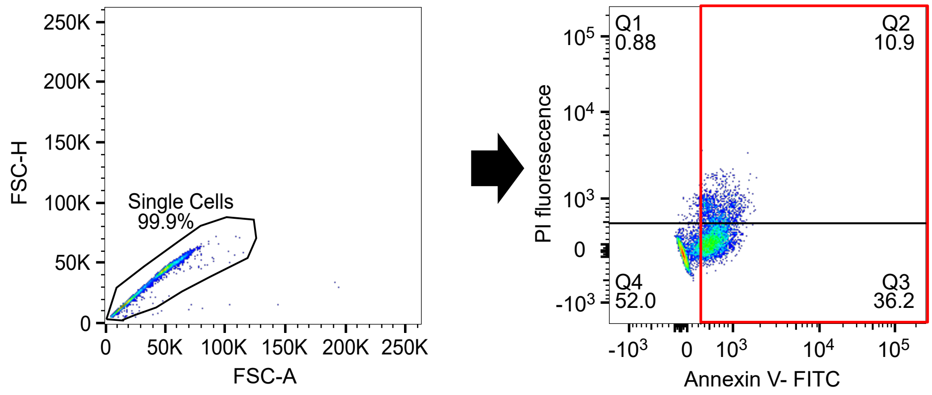

3: CLIP1-LTK-I565N

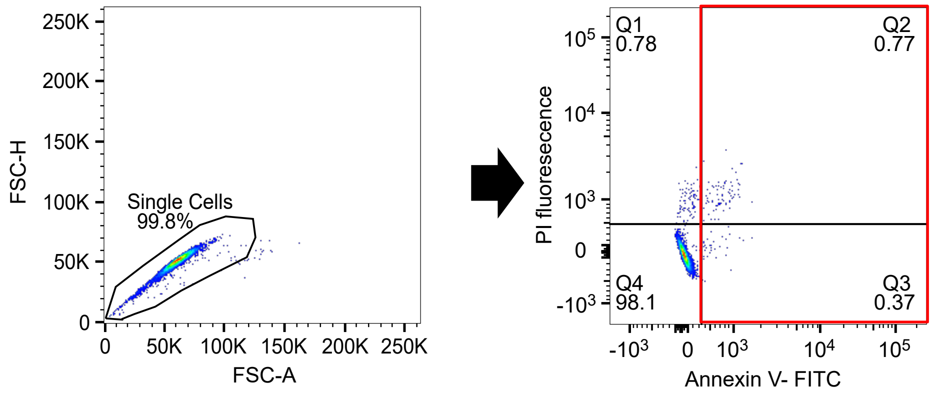

3: CLIP1-LTK-F568C

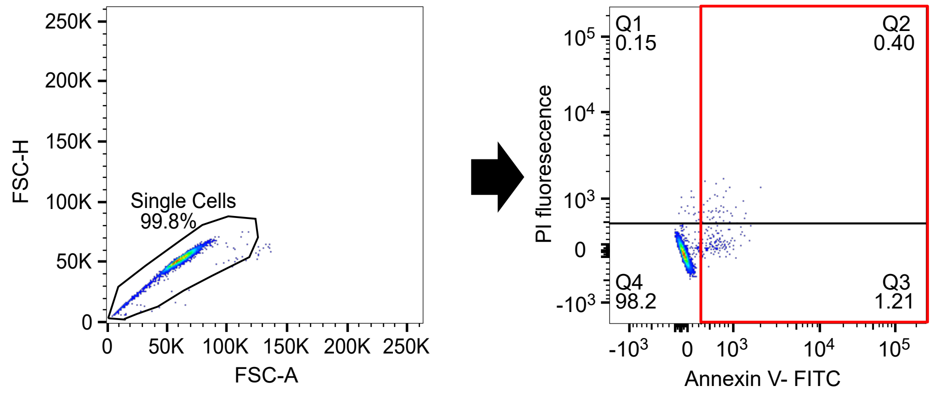

3: CLIP1-LTK-L590M

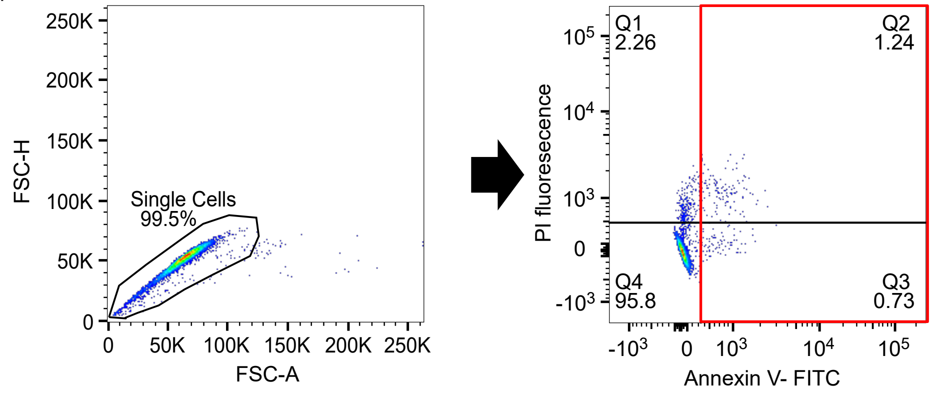

Fig.3c

3: CLIP1-LTK-L592F

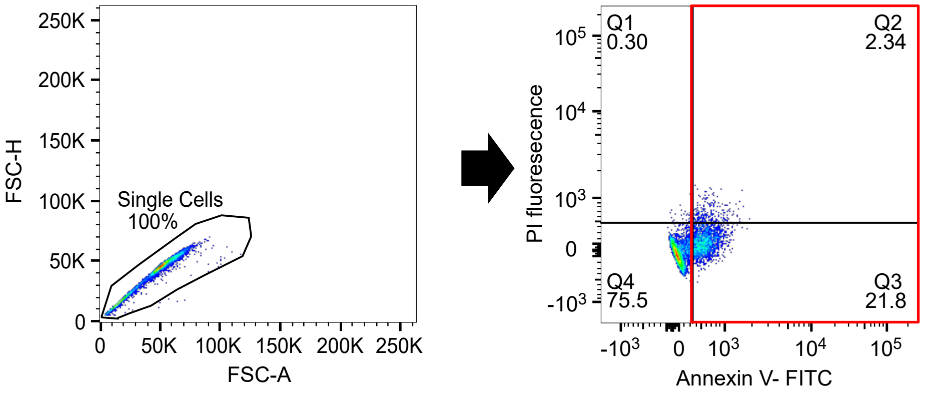

3: CLIP1-LTK-G596R

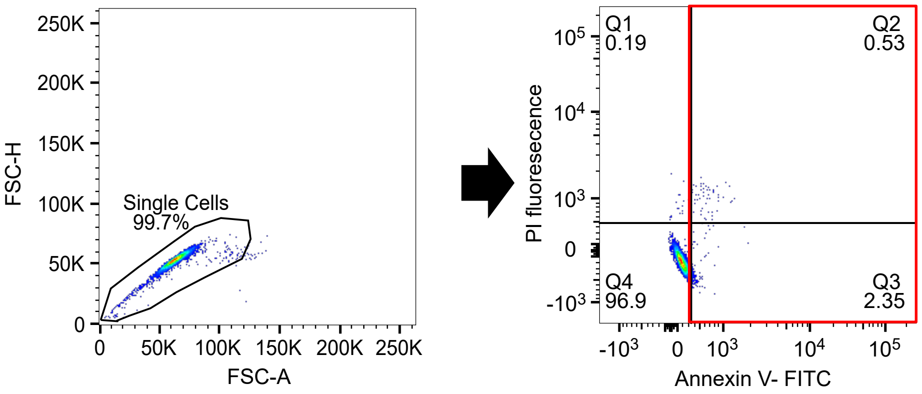

3: CLIP1-LTK-D597N

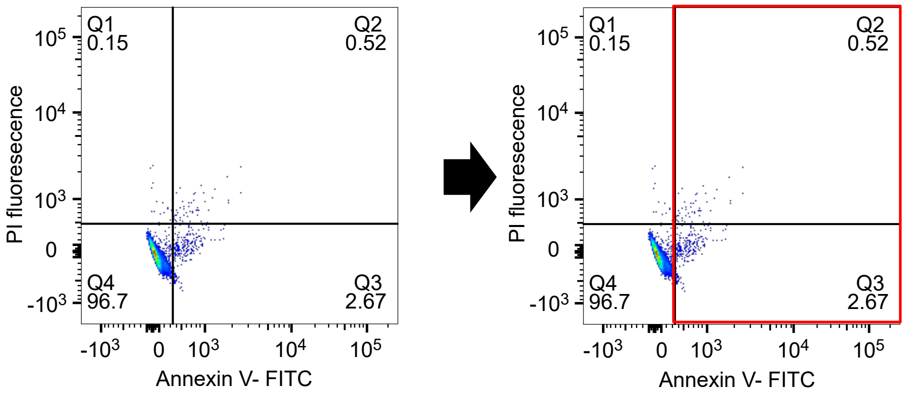

3: CLIP1-LTK-L650F

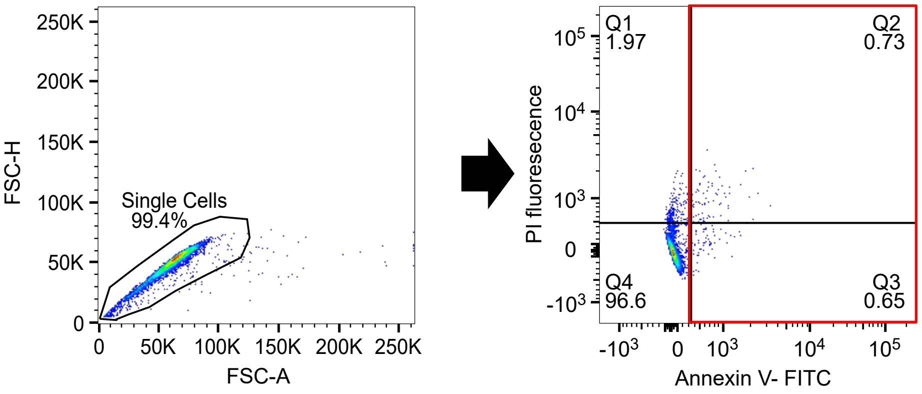

Fig.3c

3: CLIP1-LTK-G663A

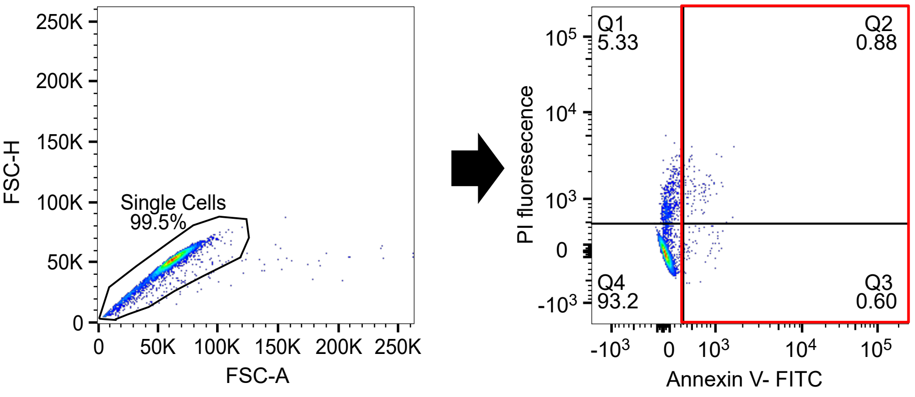

Supplement: Supplementary file 1 — Supplementary Information [file 42003_2024_6116_MOESM1_ESM.pdf]
