# Supplementary material for: LTK mutations responsible for resistance to lorlatinib in non-small cell lung cancer harboring CLIP1-LTK fusion
Source: Commun Biol. 2024 Apr 4;7:412. doi: 10.1038/s42003-024-06116-6 (PMC10995188; doi:10.1038/s42003-024-06116-6)
Supplement: Supplementary file 4 — Reporting Summary [file 42003_2024_6116_MOESM4_ESM.pdf]

Corresponding author(s): Susumu Kobayashi
Last updated by author(s): Mar 22, 2024

Reporting Summary

Nature Portfolio wishes to improve the reproducibility of the work that we publish. This form provides structure for consistency and transparency in reporting. For further information on Nature Portfolio policies, see our Editorial Policies and the Editorial Policy Checklist.

Statistics

For all statistical analyses, confirm that the following items are present in the figure legend, table legend, main text, or Methods section.

n/a	Confirmed	
☐	☒	The exact sample size (*n*) for each experimental group/condition, given as a discrete number and unit of measurement
☐	☒	A statement on whether measurements were taken from distinct samples or whether the same sample was measured repeatedly
☐	☒	The statistical test(s) used AND whether they are one- or two-sided<br>*Only common tests should be described solely by name; describe more complex techniques in the Methods section.*
☒	☐	A description of all covariates tested
☒	☐	A description of any assumptions or corrections, such as tests of normality and adjustment for multiple comparisons
☒	☐	A full description of the statistical parameters including central tendency (e.g. means) or other basic estimates (e.g. regression coefficient) AND variation (e.g. standard deviation) or associated estimates of uncertainty (e.g. confidence intervals)
☐	☒	For null hypothesis testing, the test statistic (e.g. *F*, *t*, *r*) with confidence intervals, effect sizes, degrees of freedom and *P* value noted<br>*Give P values as exact values whenever suitable.*
☒	☐	For Bayesian analysis, information on the choice of priors and Markov chain Monte Carlo settings
☒	☐	For hierarchical and complex designs, identification of the appropriate level for tests and full reporting of outcomes
☐	☒	Estimates of effect sizes (e.g. Cohen's *d*, Pearson's *r*), indicating how they were calculated

Our web collection on statistics for biologists contains articles on many of the points above.

Software and code

Policy information about availability of computer code

Data collection	Cell images were analyzed using BZ-II Viewer software (Ver.2.10). Western Blot images were captured using ImageQuant LAS4000 mini (Ver.1.00). Data for MTS assays and caspase-3/7 assay were captured using SoftMax Pro (Ver.7.10). Cells stained with annexin-V and PI were captured using FACSDiva software (v. 9.0) to evaluate the apoptosis. FACS data were analyzed using FlowJo software (v. 10.7.1).
Data analysis	Cell images were analyzed using BZ-II Analyzer software (Ver.1.42). Western Blot images were analyzed using ImageJ (Ver.1.53). MTS analyses were performed using Prism GraphPad (Ver.8.4.1). All images were assembled and figures were generated using Affinity Designer (Ver.1.10.5), and Microsoft Power Point 2016 (Ver.2108)

For manuscripts utilizing custom algorithms or software that are central to the research but not yet described in published literature, software must be made available to editors and reviewers. We strongly encourage code deposition in a community repository (e.g. GitHub). See the Nature Portfolio guidelines for submitting code & software for further information.

Data

Policy information about availability of data

All manuscripts must include a data availability statement. This statement should provide the following information, where applicable:

- Accession codes, unique identifiers, or web links for publicly available datasets
- A description of any restrictions on data availability
- For clinical datasets or third party data, please ensure that the statement adheres to our policy

The datasets generated during and/or analysed during the current study are available from the corresponding author on reasonable request.

Research involving human participants, their data, or biological material

Policy information about studies with human participants or human data. See also policy information about sex, gender (identity/presentation), and sexual orientation and race, ethnicity and racism.

Reporting on sex and gender	N/A
Reporting on race, ethnicity, or other socially relevant groupings	N/A
Population characteristics	N/A
Recruitment	N/A
Ethics oversight	N/A

Note that full information on the approval of the study protocol must also be provided in the manuscript.

Field-specific reporting

Please select the one below that is the best fit for your research. If you are not sure, read the appropriate sections before making your selection.

☒ Life sciences ☐ Behavioural & social sciences ☐ Ecological, evolutionary & environmental sciences

For a reference copy of the document with all sections, see nature.com/documents/nr-reporting-summary-flat.pdf

Life sciences study design

All studies must disclose on these points even when the disclosure is negative.

Sample size	Pilot experiments and previous published results were used to estimate the sample size such that appropriate stastical tests could yield significant results. The exaxt numbers used in the study are indicated in the respective figure legends.
Data exclusions	No date were excluded from analyses.
Replication	All experiments have been performed in at least three indipendent wells for each experiments, and performed at least three indipendent experiments.
Randomization	In all experiments, wells of equal cell numbers were randomly assigned to controls and targeted cells.
Blinding	Investigators were blinded to group a; ocation during data collection and analyses.

Reporting for specific materials, systems and methods

We require information from authors about some types of materials, experimental systems and methods used in many studies. Here, indicate whether each material, system or method listed is relevant to your study. If you are not sure if a list item applies to your research, read the appropriate section before selecting a response.

Materials & experimental systems

n/a	Involved in the study	
☐	☒	Antibodies
☐	☒	Eukaryotic cell lines
☒	☐	Palaeontology and archaeology
☐	☒	Animals and other organisms
☒	☐	Clinical data
☒	☐	Dual use research of concern
☒	☐	Plants

Methods

n/a	Involved in the study	
☒	☐	ChIP-seq
☐	☒	Flow cytometry
☒	☐	MRI-based neuroimaging

Antibodies

Antibodies used	The following antibodies were used in this study.<br>phospho ALK/LTK (pTyr1278/672) Cell Signaling Technology 6941<br>phospho Akt (pS473) Cell Signaling Technology 4058<br>phospho Erk1/2 (pT202/pY204) Cell Signaling Technology 9106<br>total LTK Abcam ab129155<br>total Akt Cell Signaling Technology 4685<br>total Erk1/2 Cell Signaling Technology 9102<br>BIM Cell Signaling Technology 2819<br>Cleaved caspase-3 (Asp175) Cell Signaling Technology 3075<br>β-actin Cell Signaling Technology 4970<br>Anti-rabbit IgG HRP-linked Secondary Antibody Cell Signaling Technology 7074<br>Anti-mouse IgG, HRP-linked Antibody Cell Signaling Technology 7076
Validation	All anitibodies were validated by and purchased from commercial vendors and were used according the manufacture`s instructions.All anitibodies from Abcam and Cell Signaling Technology were subjected to quality control testing.

Eukaryotic cell lines

Policy information about cell lines and Sex and Gender in Research

Cell line source(s)	NIH3T3 cells were purchased from ATCC. Ba/F3, WEHI, and BOSC23 cells were kindly provided by Dr.Daniel G. Tenen (Harvard Medical School)
Authentication	Cells were authenticated by the vender or depositer and no further authentication was performed in the laboratory.
Mycoplasma contamination	Testes for mycoplasma infections is routinly performed in the laboratory.
Commonly misidentified lines<br>(See ICLAC register)	No such cell lines was used in this study.

Animals and other research organisms

Policy information about studies involving animals; ARRIVE guidelines recommended for reporting animal research, and Sex and Gender in Research

Laboratory animals	BALB/cAJcl-nu/nu, 4weeks of age
Wild animals	N/A
Reporting on sex	female
Field-collected samples	N/A
Ethics oversight	All animal experiments were approved by the institutional Animal Care and Use Committee of National Cancer Center (K20-009)

Note that full information on the approval of the study protocol must also be provided in the manuscript.

Flow Cytometry

Plots

Confirm that:

☒ The axis labels state the marker and fluorochrome used (e.g. CD4-FITC).

☒ The axis scales are clearly visible. Include numbers along axes only for bottom left plot of group (a 'group' is an analysis of identical markers).

☒ All plots are contour plots with outliers or pseudocolor plots.

☒ A numerical value for number of cells or percentage (with statistics) is provided.

Methodology

Sample preparation	Ba/F3 cells expressing WT CLIP1-LTK and mutant CLIP1-LTK
Instrument	FACSCanto TM II
Software	FACS Diva 9.0 for collection and FlowJo 10.7.1 for analysis.
Cell population abundance	Purity for single cells was >90%.
Gating strategy	FACS was performed to select for cells by size through FSC-A/FSC-H gate conducted to detect single cells

☒ Tick this box to confirm that a figure exemplifying the gating strategy is provided in the Supplementary Information.
